# Analysis of Population Substructure in Two Sympatric Populations of Gran Chaco, Argentina

**DOI:** 10.1371/journal.pone.0064054

**Published:** 2013-05-22

**Authors:** Federica Sevini, Daniele Yang Yao, Laura Lomartire, Annalaura Barbieri, Dario Vianello, Gianmarco Ferri, Edgardo Moretti, Maria Cristina Dasso, Paolo Garagnani, Davide Pettener, Claudio Franceschi, Donata Luiselli, Zelda Alice Franceschi

**Affiliations:** 1 Dipartimento di Medicina Specialistica, Diagnostica e Sperimentale (DIMES), Università di Bologna, Bologna, Italy; 2 Centro Interdipartimentale “Luigi Galvani” (CIG), Università di Bologna, Bologna, Italy; 3 Dipartimento di Scienze Biologiche, Geologiche e Ambientali (BiGeA) – Sezione di Biologia, University of Bologna, Bologna, Italy; 4 Dipartimento ad Attività Integrata di Laboratori, Anatomia Patologica, Medicina Legale – U.O. Struttura Complessa di Medicina Legale, Azienda Ospedaliero – Universitaria di Modena, Modena, Italy; 5 Coordinación Nacional de Control de Vectores, Córdoba, Argentina; 6 Centro de Investigaciones en Antropologia Filosofica y Cultural (CIAFIC), Buenos Aires, Argentina; 7 Centro Universitario Interdisciplinario sobre la Enfermedad de Chagas (CUNIDEC), BuenosAires, Argentina; 8 Dipartimento di Storia Culture Civiltà (DiSCi), University of Bologna, Bologna, Italy; University of Cambridge, United Kingdom

## Abstract

Sub-population structure and intricate kinship dynamics might introduce biases in molecular anthropology studies and could invalidate the efforts to understand diseases in highly admixed populations. In order to clarify the previously observed distribution pattern and morbidity of Chagas disease in Gran Chaco, Argentina, we studied two populations (Wichí and Criollos) recruited following an innovative bio-cultural model considering their complex cultural interactions. By reconstructing the genetic background and the structure of these two culturally different populations, the pattern of admixture, the correspondence between genealogical and genetic relationships, this integrated perspective had the power to validate data and to link the gap usually relying on a singular discipline. Although Wichí and Criollos share the same area, these sympatric populations are differentiated from the genetic point of view as revealed by Non Recombinant Y Chromosome genotyping resulting in significantly high Fst values and in a lower genetic variability in the Wichí population. Surprisingly, the Amerindian and the European components emerged with comparable amounts (20%) among Criollos and Wichí respectively. The detailed analysis of mitochondrial DNA showed that the two populations have as much as 87% of private haplotypes. Moreover, from the maternal perspective, despite a common Amerindian origin, an Andean and an Amazonian component emerged in Criollos and in Wichí respectively. Our approach allowed us to highlight that quite frequently there is a discrepancy between self-reported and genetic kinship. Indeed, if self-reported identity and kinship are usually utilized in population genetics as a reliable proxy for genetic identity and parental relationship, in our model populations appear to be the result not only and not simply of the genetic background but also of complex cultural determinants. This integrated approach paves the way to a rigorous reconstruction of demographic and cultural history as well as of bioancestry and propensity to diseases of Wichí and Criollos.

## Introduction

The genetic structure of a population is shaped by its evolutionary history that encompasses all the past demographic events and the complex gene-culture coevolution [1]–[3].

Genes and culture evolve at different rates and under different rules, but at the same time they are in a close interaction. The dynamic equilibrium between cultural and molecular anthropology has been scarcely investigated so far, mainly due to the fact that the two subjects traditionally had a very low level of interplay. This aspect becomes even more striking when considering host-pathogens interaction, in particular with health emergencies in developing countries. A paradigmatic example is constituted by Chagas disease, which is endemic in almost all the South American continent [4].

The Chagas disease is caused by the infection of *Trypanosoma cruzi*, and a study on 283 human mummies demonstrates that the sylvatic cycle (animal infected) of Chagas was likely established at the very first time of human colonization (Chinchorro culture) of the Andean coast [5]. This result implies that *T. cruzi* and humans coevolved for more than 9000 years consistently shaping the genetic and immunological background of native populations. In such a framework it is likely that south Amerindians have developed a peculiar way to cope with *T. cruzi* infection and this has to be taken into account in Chagas disease epidemiology and treatment strategies. Therefore when dealing with South American populations, which have complex evolutionary histories [6]–[9], marked by genetic bottlenecks and different degree of intermixing between Indigenous American, European and African populations [10]–[11] the ability to disentangle the genetic structure of south American populations is essential.

Sub-population structure, the intricate kinship dynamics and the incompleteness and unreliability of the birth, marriage and death registers might introduce biases in such studies and could invalidate the efforts to understand and challenge Chagas and other diseases in these geographical areas.

In Misión Nueva Pompeya, a community in the inner part of the Gran Chaco region in Argentina, the Native American Wichí cohabit with the admixed population generally named Criollos, composed by historical intermarriages between various Chaquean populations and first European male colonizers. The two populations dwell the same geographical environment and the same social space, but they are deeply different in terms of cultural and ethnographical structure. As they belong to different cultural traditions they do not share cultural rules, but in the real practice they are mixed creating a complex society stratification that only the genetic analysis can clarify. Identity, in these populations, is a matter of decision and social affiliation, so defining oneself as Wichí and Criollo has strong fallouts, from the economic, political and social point of view. To be aborigines in Misión Nueva Pompeya has indeed strong consequences both on a social level and in respect to the national government, the most important of which is to have rights upon the land. On the other hand the Criollos are the ones who have access to political decision making, because they have higher degree of education and a considerable knowledge of the law and the rules [12].

Usually human population genetic studies analyze a sample of putative unrelated individuals living in a small geographic area, but the risk of using such sampling strategy in Misión Nueva Pompeya is to bundle both native and admixed individuals into a unique population. To prevent this bias our sampling strategy started on a field work based on a detailed knowledge of family relationships, demographic patterns, social and cultural behavior and was based on a large sample, likely the entire population of the area of Misión Nueva Pompeya.

In order to understand and clarify the pattern of distribution and morbidity of Chagas disease in Wichí and Criollos [13] and possibly to detect marks of evolutionary adaptation to *T. cruzi,* we decided to follow an innovative bio-cultural model in population genetic studies, taking into account the complex cultural interactions between them [12]. The aim of the present study was to reconstruct a) their genetic background and structure, b) the pattern of admixture at a population level, c) the correspondence between genealogical and genetic relationships. This double integrated perspective has the power to validate each other, to check the meaning of the results and to link the gap that usually relies on a singular discipline.

### Wichí, Criollos and their Geographical Context

**Figure 1:**
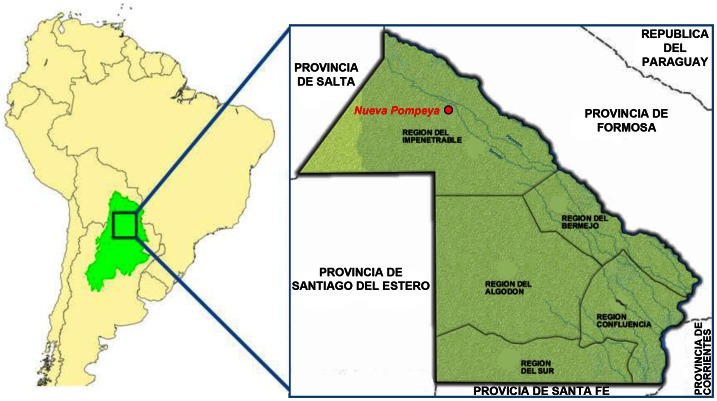
Map of Gran Chaco region and the focus on Chaco province in Argentina with highlighted Misión Nueva Pompeya where samples were collected.

Aboriginal people from Gran Chaco comprises 16 living ethnic groups, clustered in six linguistic families (Matako-Maka, Guaykurù, Lule Vilela, Tupi Guarani, Lengua Maskoi, Zamuco) [14]. They were seasonal hunter-gatherers living in small groups now generally settled in villages, and their phenotypic diversity is complex, matching only partially their linguistic structure. Misión Nueva Pompeya is a village in Chaco lowland dry forest known as “El Impenetrable” (The Impenetrable), located in the northwest of the province of Chaco, Argentina. In this geographic area cohabit two different populations (Wichí and Criollos) settled in several rural communities called *parajes* (Figure 1). Wichí are indigenous hunter-gatherers, speaking a language that belongs to the Matako-Maka family, living in central-western province of Chaco and also in north-eastern Salta, between the Bermejo and Pilcomayo rivers. These groups lived for long time without contacts with Europeans as the Spanish process of colonization fundamentally concentrated on the north-eastern region of Argentina, then inhabited by sedentary populations with strong Andean influences. The populations of the Chaco have been traditionally characterized by a livelihood tied to hunting, fishing and gathering, activities divided according to gender and age group. Today, economic activity has undergone deep changes due to the deforestation of many wooded and forested areas, the salaried work and the social welfare plans. Even the schooling of the youth of both sexes carried out some severe consequences for the cultural and economic uses, as well as implied changes on medical and sanitary systems. Wichí share the geographical environment with local Criollos populations. Criollo, typically defined as “the child of a European father born in any part of the world, except for Europe”, also supposed a mixed origin that can be European or even Native but deriving from inland regions less affected by the recent European immigration [15].

The Criollos of Chaco descend from families settled at the borders of the region, in the land that nowadays is defined as province of Salta and Santiago del Estero, since the 16^th^ and the 17^th^ centuries, moving later into the dry forest to better feed their cattle. This event took place between the 19^th^ and 20^th^ centuries, thus affecting aboriginal lands. Criollos are the descendants of the first “mestizo” inhabitants of the Hispano Quechua Andean area of the north east, who later expanded and colonized the region in the early 20^th^ century. Also called “chaqueños”, they are the descendants of those who first populated the North West area of the Andes – an area of Hispanic-Quechua roots – and who then spread and colonized the region at the beginning of the 20^th^ century [16].

## Materials and Methods

### Sampling Strategy

Current Criollos are really mixed with Wichí due to hidden interaction along decades; phenotypic traits are fused with Wichí and morphological differences are in many cases undetectable. A traditional sampling approach could involve both native and admixed individuals, considering them as a meta-population.

In order to understand which is the influence of the genetic structure on the prevalence of Chagas infection in these two sympatric populations, we sampled, after a long-term fieldwork carried out by cultural anthropologists, not only a sample of putative unrelated individuals from the two groups, but likely the entire population of the area. 552 subjects (342 Native Wichí and 210 admixed Criollos) were then recruited on the basis of their self-reported ethnicity. The great number of samples collected represents almost the whole of *parajes* population in the panorama of Misión Nueva Pompeya. The strategy to collect all the individuals and not just a representative subgroup of the two populations will enable the identification of the actual population structure that normally remains underestimated in genetic studies.

Whole blood samples were taken from venous blood in adults (the serum was separated and stored at −20°C until use) and capillary blood in children (stored in buffered glycerine at room temperature, Serokit).

### Ethics Statement

The sampling was in accordance with the ethical standards for human experimentation and with the Helsinki Declaration of 1975, as revised in 1983. The study was approved from the ethical committees of the University of Bologna (Azienda Ospedaliero-Universitaria di Bologna - Policlinico Sant'Orsola-Malpighi) and of the University Hospital of Maternity and Neonatology, Universidad Nacional de Córdoba, Argentina. The Ministry of Health of the province of Chaco provided further authorization to perform the survey. People who voluntarily participated to the study, were previous informed of its objectives through autochthonous translators and then they signed the consent form written in Spanish and Wichí languages.

### Genealogical and Demographic Analyses

A deep study on demographic data and their revision was preliminarily performed on field. Genealogies of all the *parajes* inhabitants were reconstructed by the cultural researchers during six years of fieldwork (2004–2010). Beside the usual genealogical diagrams taken for a population, specific questionnaires were developed and employed in both languages aiming at obtaining a wide report upon the genealogical memory of the two populations (concerning remembrances of names, ethnic identities, birthplaces, socio-cultural habits, Chagas disease perception, etc.). Furthermore data on cultural, economic and social-health conditions were collected in order to reconstruct the social panorama of the population of Misión Nueva Pompeya. Data collected on serological prevalence of Chagas disease were reported in previous study [13] on the basis of self reported ethnicity affiliation.

All the participants were identified by anonymized codes. The genealogies of Wichí and Criollos individuals were reconstructed using detailed self reported information obtained during the fieldwork and by the deep analysis of pedigrees, combining with data coming from official sources of demographic registry (CENSUS). A pedigree for each paraje was drawn using GenoPro® 2007 v. 2.0.1.6 (http://www.genopro.com) and was integrated with male and female lineage data obtained from the genetic analysis.

### Molecular Analysis

DNA was extracted from 2 mL whole blood using the QIAamp DNA Blood Midi Kit® (QIAGEN). All DNAs were then quantified by fluorometric dsDNA assay using PicoGreen® (Quant-iT™ PicoGreen® dsDNA kit, INVITROGEN), and normalized to a concentration of 100 ng/µl.

The Non Recombining Y chromosome (NRY) genetic variability was tested by 16 SNPs and 17 STRs in the 243 males of the total sample. Details on NRY analysis methods are provided in Text S1 and in Tables S1 and S2.

For the mitochondrial haplogroup classification, all the available 552 samples were analysed using the methods described in Text S2. Haplogroup nomenclature followed the most recently updated versions of the Native American phylogeny given in [17]–[20], and the most-recent phylogenetic data on global human mtDNA variation were taken into account [21].

### Statistical Analyses

Basic parameters of molecular diversity and population genetic structure, including analyses of molecular variance (AMOVA), were calculated using the Arlequin package version 3.5 [22]. The statistical significance of Fst values was estimated by permutation analysis, using 10,000 permutations. Fst values were calculated according to the number of pairwise differences between sequences (for mtDNA) or haplogroups (for the NRY). To test selective neutrality and potential departures from a null hypothesis of mutation-drift equilibrium and constant population size, the Tajima’s D was used.

For the admixture estimation we applied two different methodologies implemented in Admix 2.0 [23]–[24] and Admix 95 [25] softwares, using the parental populations reported in Table S3.

Median joining (MJ) networks [26] for the entire dataset (NRY and mtDNA) were built using the software NETWORK v 4.510 (http://www.fluxus-technology.com).

For NRY we assigned weights, inversely proportional to the STR variance, ranged from 1 to 10. For mtDNA we adopted the mutation weights as described in [27] and a median weight for the not reported mutations, taking into account the improved molecular clock for dating human mtDNA as proposed by [28]. Non metric multidimensional scaling (nmMDS) and cluster analysis were carried out by R package (http://www.r-project.org/), using Fst pairwise matrices both intra and inter populations.

## Results

### NRY Genetic Variability

**Figure 2:**
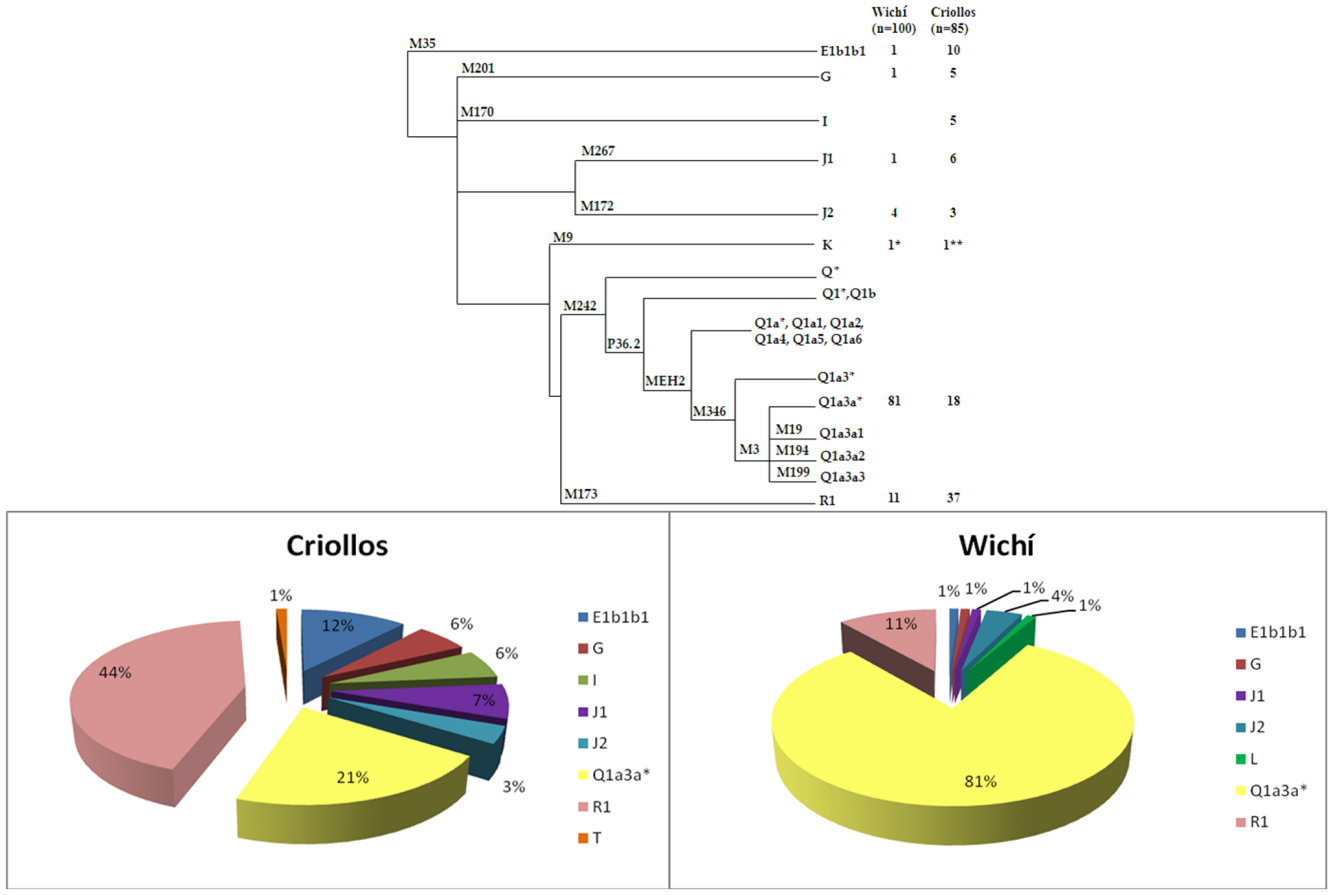
Phylogenetic tree with haplogroup frequencies and corresponding typed mutations in Wichí and Criollos. All Amerindian lineages are derived from M242 marker and were typed by the novel Q multiplex. *Inferred as L with Bayesian approach **Inferred as T with Bayesian approach. The inferences are evaluated by Haplogroup predictor (www.hprg.com/hapest5/).

Within the 243 males of Misión Nueva Pompeya we identified by NRY biallelic markers genotyping and by STRs haplotypes, 184 unrelated individuals (100 Wichí and 84 Criollos). As expected NRY haplogroup diversity is extremely low (0.3331+/−0.0579) in Wichí compared to Criollos (0.7461+/−0.0366), due to the presence in the latter of 80% of European ancestry haplogroups, subdivided in R1 (44%), E1b1b1(12%), G and I (both 6%) and J1 (7% ) and J2 (3%) lineages (Figure 2). However even among Wichí individuals we found nearly 20% of European lineages, mostly differentiated in R1 (11%) and J2 (4%) haplogroups.

The genetic analysis of the Amerindian NRY highlighted the absence of the Q1a3* paragroup (M346) and only the presence of Q1a3a* (xM19xM194xM199) with frequencies of 80% and 20% in Wichí and Criollos respectively.

STRs genotyping in Wichí population identified 58 different haplotypes (H = 0.9622+/−0.0118) with a strong founder effect in the Native Q1a3a* lineage, where 17 individuals share the same haplotype. A complete list of the NRY haplotypes is reported in Table S4. Higher values of haplotype diversity (H = 0.9905+/−0.0038) and evidence of higher genetic variability at each locus (θH) are reported for Criollos in Figure S1.

**Figure 3:**
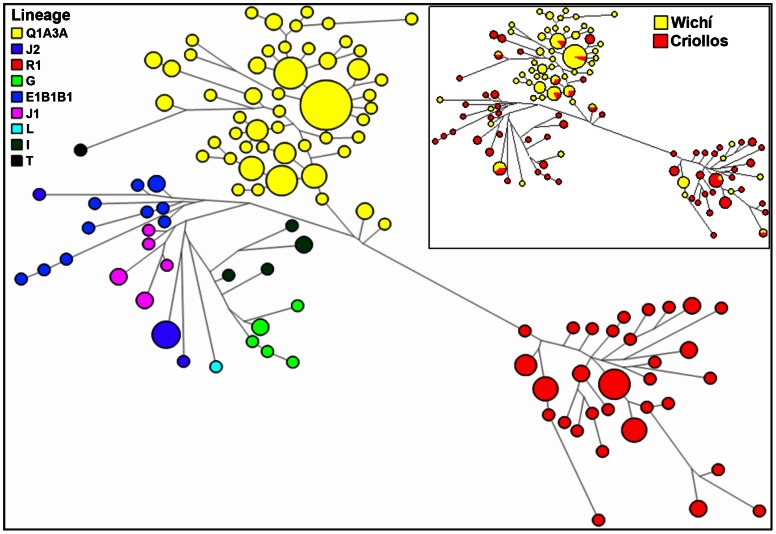
Median joining network for the total Wichí and Criollos samples.

Relationships between haplotypes in the two investigated populations were analyzed by Median Joining Network (MJN) analysis (Figure 3), which emphasizes a clear separation between the two populations with some limited overlapping areas (top right of the figure) and a sharp subdivision within the different NRY haplogroup clusters. The split between European and Amerindian haplogroups almost corresponds with Wichí and Criollos chromosomes distribution, mirroring the almost complementary admixture histories of the two populations.

**Figure 4:**
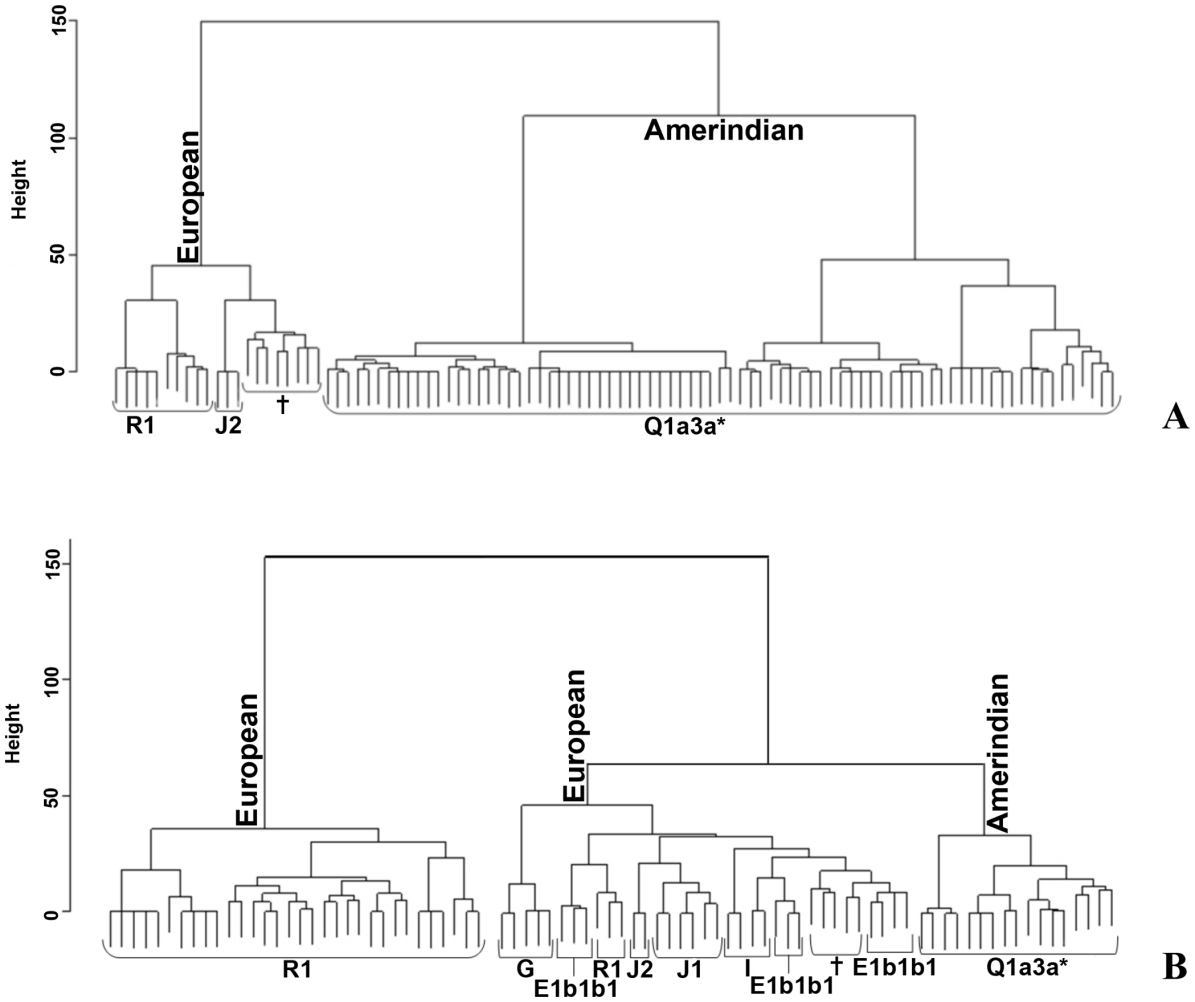
Cluster analysis in Wichí (A) and Criollos (B) based on 17 Y-STRs. **†These clusters include some different haplogroups.**

A cluster analysis (Figure 4) based on 17 STR loci supported a strong split in two main branches, Amerindian and European ones, with a typical comb-like structure for Wichí (Figure 4A) and a deeper configuration characterizing the most ancient European lineages in Criollos (Figure 4B).

In order to measure the extent of the different genetic pattern observed in Wichí and Criollos, AMOVA was carried out considering STRs and SNPs data. Both the results (Fst = 0.16232, p<0.01 for STRs and Fst = 0.31916, p<0.01) highlighted a significant differentiation between the two populations, in agreement with the self-identity information collected on the field.

To assess the degree of admixture in the two Gran Chaco populations, we collected from the literature a list of SNPs and STRs allele frequencies of the putative ancestral populations from South America and Europe (Table S5). Wichí group confirms a higher proportion of Amerindian ancestry, more than 90% (estimated by Admix 2.0), while in Criollos ranges between 20–25% (STRs and SNPs data respectively).

In order to dissect the European component in this admixed group we used two STRs haplotype datasets from Italian and Iberian populations. The results underlined the major Iberian contribution (>50%) and a comparable contribution by Italian and Amerindian ancestral populations (26% and 22% respectively, Table 1).

**Table 1:** Admixture in Wichí and Criollos populations.

Analysis	Marker		Parental		
			Amerindian	European	
**Admix2.0**	SNPs	Wichí	0.925±4.0	0.75±4.0	
	SNPs	Criollos	0.252442±0.0589801	0.747558±0.0589801	
	STRs	Criollos	0.203524±0.0452936	0.796476±0.0452936	
			**Amerindian**	**Italian**	**Iberian**
**Admix95**	STRs*	Criollos	0.2211±0.0035	0.2608±0.0049	0.5181±0.0076
	STRs**	Criollos	0.2098±0.0068	0.2076±0.0256	0.5826±0.0268

* 1 Subset: DYS19,DYS389I,DYS389II,DYS390,DYS391,DYS392,DYS393. R^2^∶0.999956.

** 2 Subset: DYS437, DYS438, DYS439, DYS448, DYS456, DYS458, DYS635. R^2^∶0.999636.

**Figure 5:**
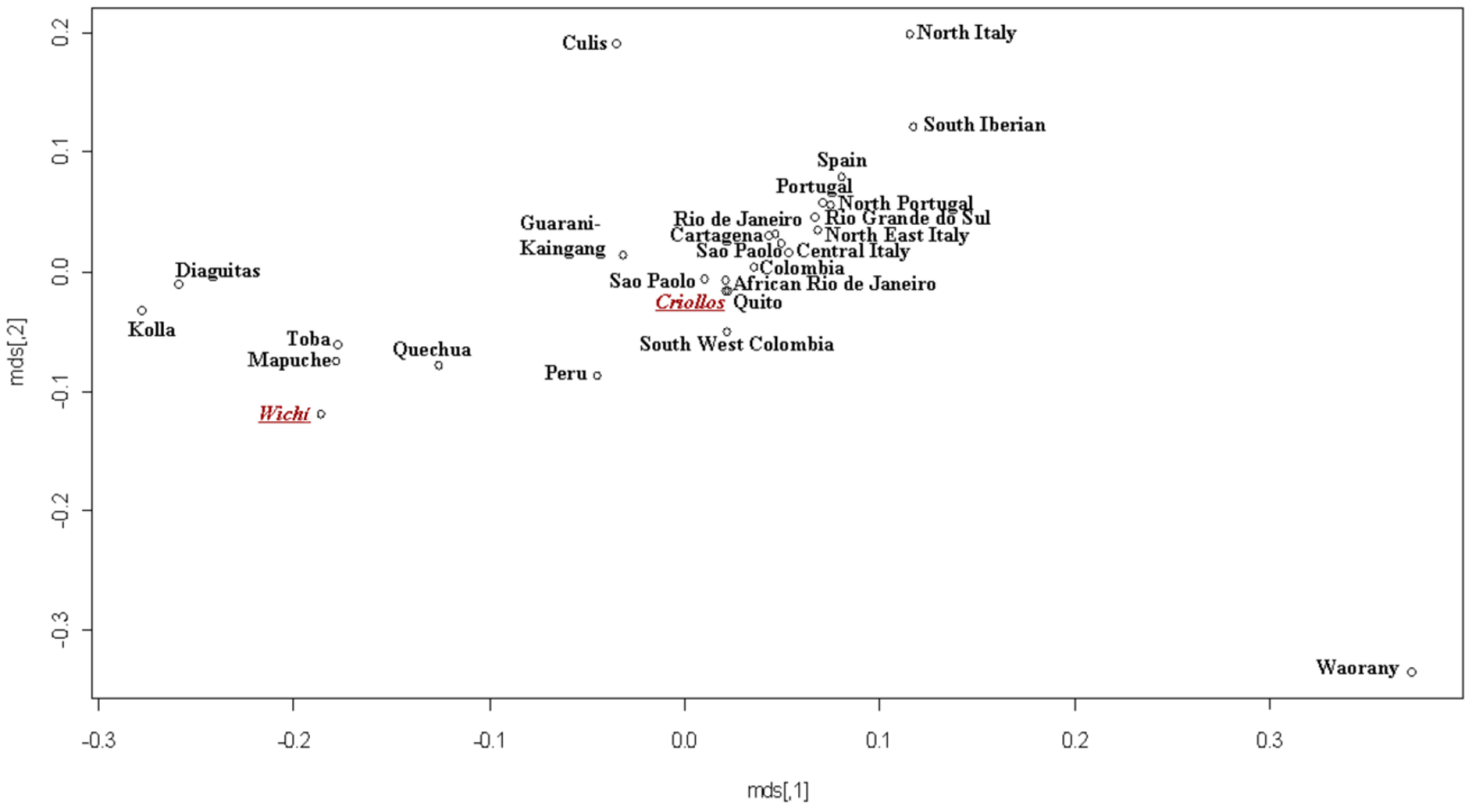
MDS performed by a Slatkin matrix of distances computed by 12 Y-STRs loci in American and European comparison populations (considering only Wichí and Criollos unrelated samples).

**Figure 6:**
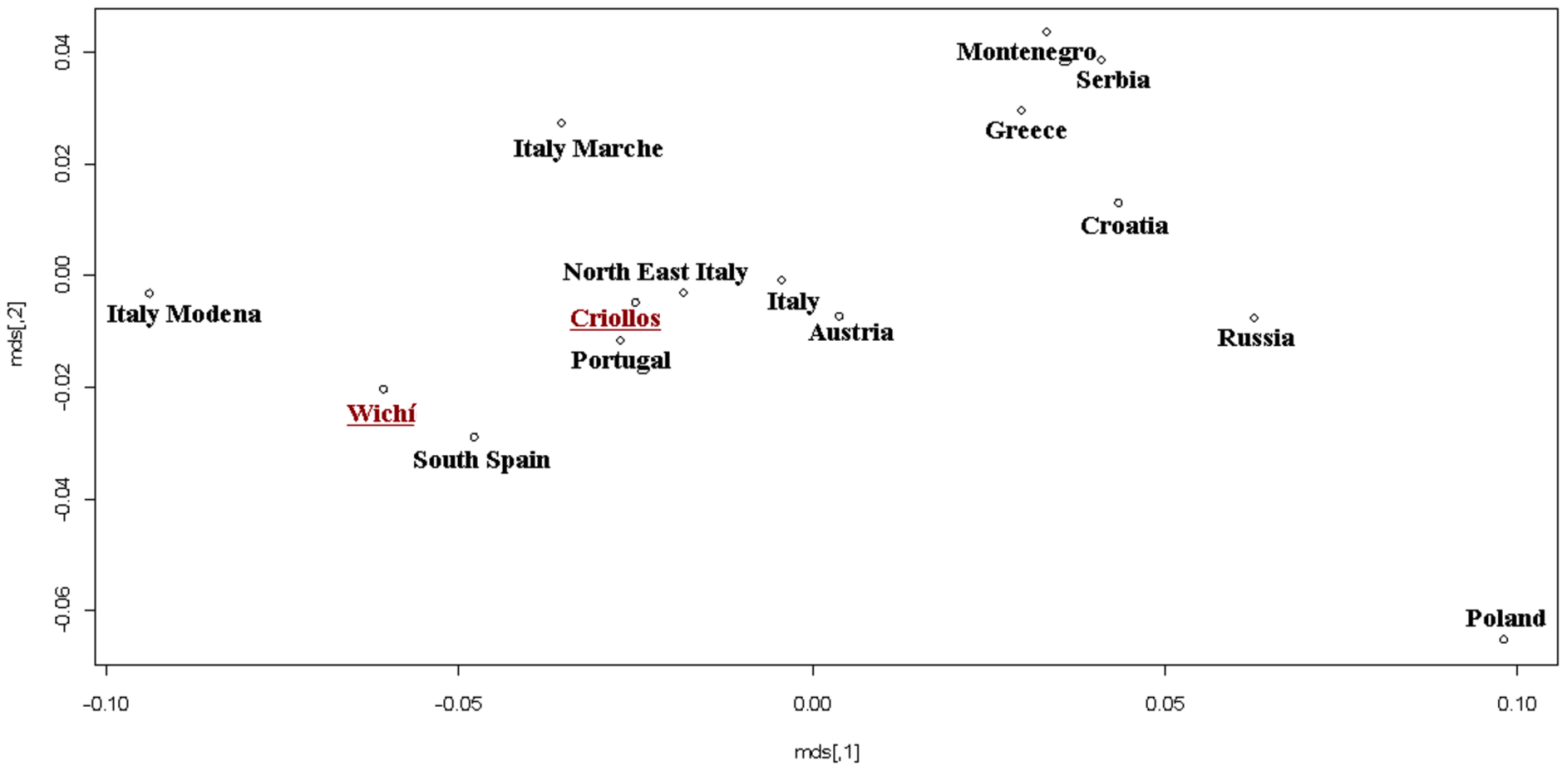
MDS performed by a Slatkin matrix of distances computed by 17 loci on 13 European populations with European Wichí and Criollos haplotypes.

Moreover, with the aim to disentangle the genetic structure of the two populations, a nmMDS based on a Slatkin matrix was performed considering the total Wichí and Criollos samples (data not shown) and the unrelated sample subsets (Figure 5) using 12 STRs data in 26 different populations of European and Amerindian ancestry collected from the available literature (detailed references in Table S3). Similar results were obtained for both the comparisons, Wichí in the cluster with native people from Argentina (Toba, Kolla, Diaguita, Mapuche) and Criollos within a wide and scarcely diversified cluster including American admixed populations (from Ecuador, Brazil, Columbia) and European ones (from Portugal, Spain and Italy). Using 17 STRs loci and focusing on the European component, a more detailed pattern emerged, with Wichí clustering closer to Southern Spanish and Criollos populations than to Portuguese and Italian ones (Figure 6).

Furthermore MJN analysis allowed us to better investigate the relationships between native Q haplotypes in Wichí and Criollos and other South American populations (Figure S2). We found that both Wichí and Criollos share haplotypes almost exclusively with Toba from Gran Chaco and with no other Amerindian population of the ones we had available.

### mtDNA Genetic Variability

The observed variability of the mitochondrial control region revealed that 99,3% of our samples belong to 4 out of the 5 Native American haplogroups (A4, B4, C1 and D4). Furthermore of the 106 different haplotypes we found (Tables S6 and S7), 36% were exclusively present in Wichí, 51% in Criollos and 13.20% shared by both groups. Aiming to test our sampling strategy, we considered, as for NRY, the total and the unrelated samples to estimate the genetic diversity. Wichí have lower gene diversity in unrelated sample than in total population (shifting from 0.8261 to 0.8434 respectively), whereas in Criollos occurs the contrary (shifting from 0.9784 to 0.9562) (Table 2). Nucleotide diversity (π) within population is in good agreement with values reported by the literature for the Criollos component [29]–[30] (Tables S6 and S7), whereas it is quite lower among Wichí.

**Table 2:** mtDNA haplogroup frequencies in South America populations available in literature and relative references.

Population	N	A4	B4	C1	D4	Other	Reference
**Amerindians**	64	9.2	64.6	16.9	6.2	-	Dipierri et al., 1998
**South America**	1184	14.7	42.2	28.6	14.5	-	Garcia and Demarchi, 2009
**North Argentina**	98	21.4	11.2	21.4	12.3	33.7	Bobillo et al., 2009
**Gran Chaco**	240	10.8	47.9	11.3	28	2.0	Demarchi et al., 2001
**Wichí**	129	8.5	54.3	8.5	27.1	1.6	Torroni et al. 1993, Bianchi et al. 1995, Demarchi et al. 2001

**Figure 7:**
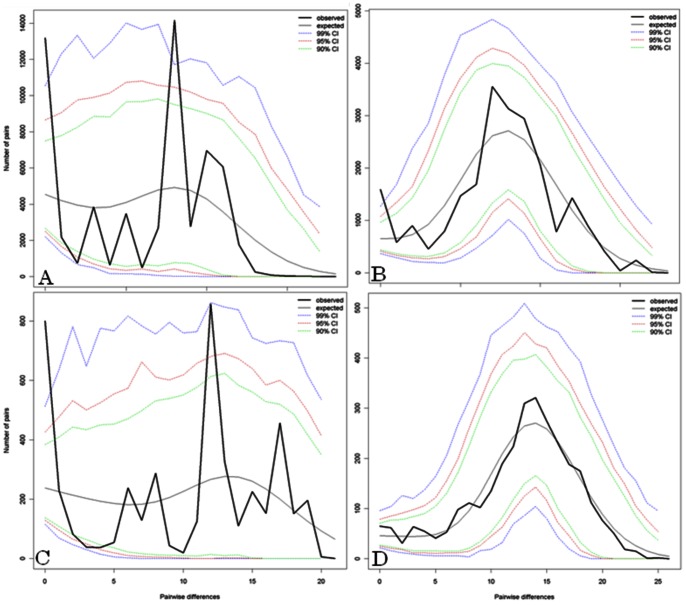
Mismatch distributions as revealed by the demographic expansion model. In A and C Wichí estimates are shown, whereas B and D represent the Criollos ones. First row is based on the total sample, the second one on the unrelated samples.

To test selective neutrality and potential departures from a null hypothesis of mutation-drift equilibrium and constant population size we performed Tajima's D considering total and unrelated Wichí and Criollos samples (Table S8). A negative Tajima's D was found in Criollos samples and a positive value in Wichí. In agreement with this analysis, mismatch distribution revealed different and opposite trends in the two populations (Figure 7). Wichí population is at demographic equilibrium showing ragged multimodal curves (Figures 7A and 7C), reflecting an ancient settlement (for unrelated individuals: HR-i = 0.07, p<0.05); on the contrary, the smooth unimodal curve shown by Criollos (Figures 7B and 7D) suggests a demographic expansion in good agreement with South-America population history (for unrelated individuals: HR-i = 0.003, p = 0.81).

**Figure 8:**
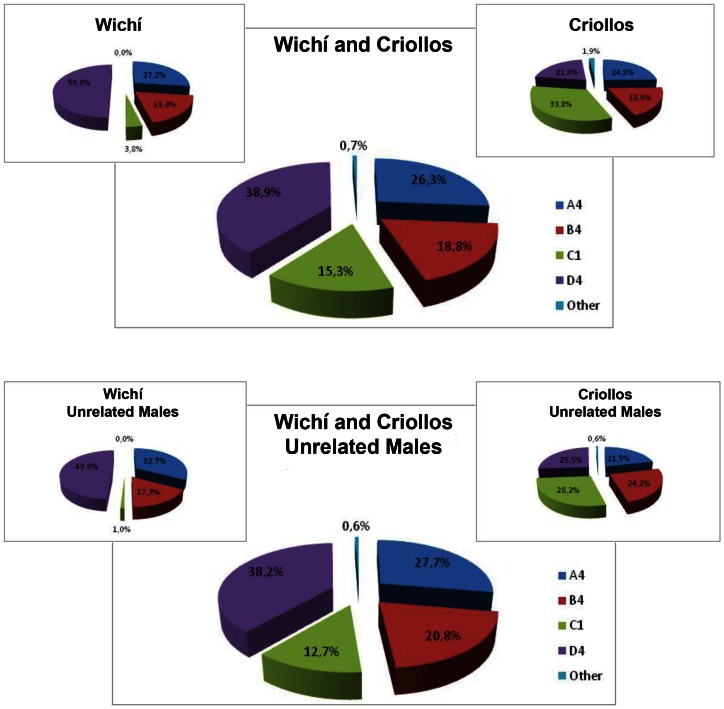
mtDNA haplogroup frequencies for Wichì and Criollos populations presented for whole sample (A) and only unrelated males (B).

Fst genetic distances for sequences data and haplogroup frequencies confirmed a significant differentiation between Wichí and Criollos (Fst = 0.13028 and 0.10695; p-value <0.01, respectively), although more weakly than the male perspective. In fact the haplogroup frequencies distributions between Wichí and Criollos are statistically significant (p<0.01), considering both total and unrelated samples (Figure 8 and Table 3). It is noteworthy that only 0.7% of the maternal lineages are of European ancestry and that 50% of Wichí individuals belongs to the D4 haplogroup.

**Table 3:** Gene diversity (H) of the two populations presented as total and unrelated samples.

Population	Haplotypes (N)	H
**Wichí Total**	52	0.8434+/−0.0127
**Criollos Total**	64	0.9562+/−0.0064
**Wichí Unrelated**	22	0.8261+/−0.0251
**Criollos Unrelated**	42	0.9784+/−0.0059

Inter-populations comparisons with five other samples from South American available in literature (Table 2), exhibited in the two groups the highest incidence of A4 and D4 lineages and the lowest of B4 haplogroup.

**Figure 9:**
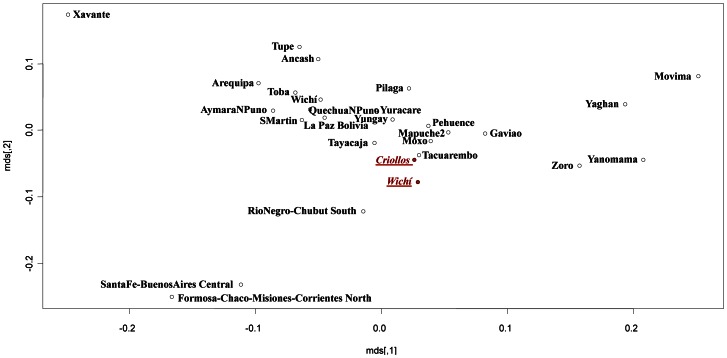
MDS performed by a Slatkin matrix of distances representing total Wichí and Criollos in comparison to different populations from South America.

**Figure 10:**
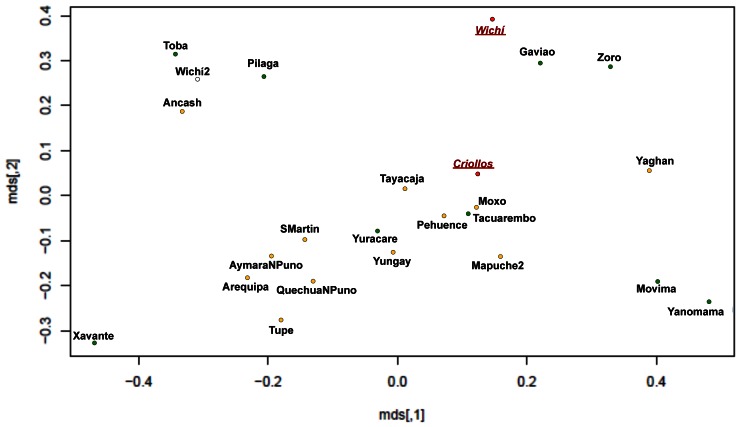
MDS performed by a Slatkin matrix of distances representing unrelated males Wichí and Criollos in comparison to Amerindian populations. In green and in orange are highlighted Amazonian and Andean populations respectively, while Wichí2 unknown population is white colored.

Slatkin’s genetic distance matrix was used to perform nmMDS. Two main clusters are highlighted, the first gathering the majority of Amazonian populations and the second the bulk of the Andean populations. The analysis carried out on the total Wichí and Criollos individuals (Figure 9) showed that the two groups cluster together, near the Andean groups, and contrarily to what observed for NRY, separated from Toba population. However taking into account only the unrelated Wichí and Criollos and excluding the Argentinean admixed populations, a different and more clear pattern (Figure 10) was shown, underlining Criollos and Wichí separated from each other and clustering within the Andean and the Amazonian groups respectively. It is remarkable that another Wichí sample collected in a previous study [29] clusters near to Toba population and apart from our sample.

**Figure 11:**
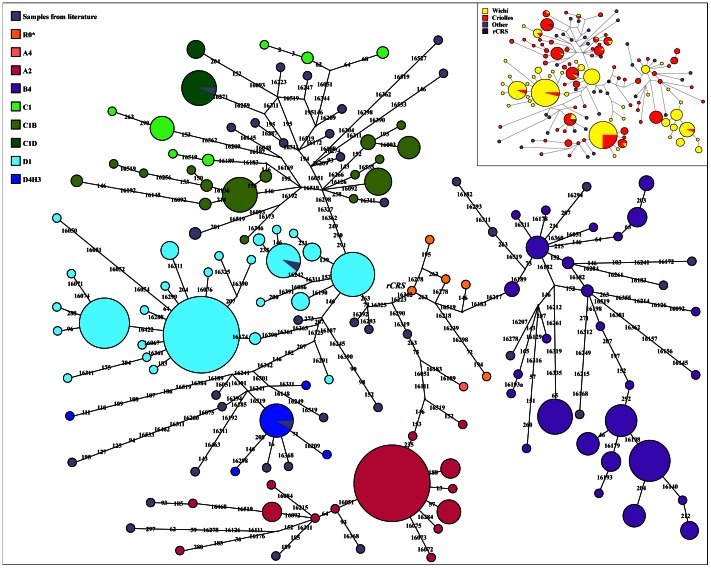
Median Joining Networks showing the mtDNA variability among the samples and, in little, the relationships among all Wichí and Criollos mitochondrial haplotypes. Grey circles represent publicly available sequences (one for each subhaplogroup, as described in Table S9 supplementary materials) not proportional to the observed dimension of the haplotypes; they were added in order to better define the topology of the network as they represent the lineages defined until now.

A MJN based on d-loop sequence from nucleotide 16047 to nucleotide 300 was performed, considering all the populations included in Table S9. For these populations we used only one haplotype representative of main sub-haplogroups (Figure 11). The resulted pattern displays a clear differentiation among the four Native Amerindian macro-haplogroups with a minimum overlapping between Wichí and Criollos. A strong founder effect for A4 (including A2 haplogroup) and D1 macro-lineages is observed, whereas B4 and C1 haplogroups show a more diversified sub-lineages, indicative of an ancestral state that underwent expansion. In particular, C1 lineage is typical of Criollos with a very little haplotype sharing with Wichí. Within D4 lineage the separation between D1 sub-haplogroup and the rare branch D4h3 (in the lower part of the D4 cluster) is mainly due to the Criollos component, as suggested also by the gene diversity values (H) for this haplogroup (H = 0.66±0.03 for Wichí, H = 0.84±0.03 for Criollos). Inside this branch, multiple clusters define lineages peculiar to one of the two populations.

### Linking Cultural and Molecular Results

In order to test the effectiveness of our sampling strategy and of the proposed integrated anthropological model, we compared kinship trees obtained from data retrieved by the cultural fieldwork with those from genetic data. To perform this comparison and to test the reliability of the model, we considered one of the most studied and well characterized families in our sample set.

**Figure 12:**
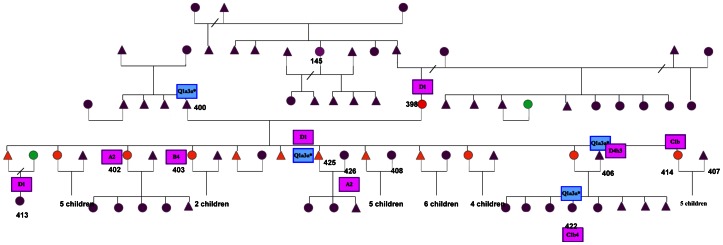
Reconstruction of a genealogic tree belonging to Wichí population. Triangles and circles represent males and females; blue and pink labels represent NRY and mtDNA haplogroup classification. In red the case of ID398 and in orange her children on the basis of the self-reported identity. A case of intergenerational union is also reported (in green the same individual is shown).

The integration of male and female lineages data in the genealogical trees reconstructed by fieldwork marked some inconsistencies (see Figure 12).

From the maternal perspective, in particular, the mismatch between the genetic results and the reconstructed relationships based on “self-reported identity” has revealed that ID398 (Wichí) despite having twelve children (six males and six females, in Figure 12) is not the biological mother of any of the five (three females and two males) that we have analyzed for genetic markers. Despite both ID398, the mother, and ID425, the child, belong to the D1 haplogroup, they show two different haplotypes, mtHt15 and mtHt4 respectively (Table S6). These two D1 haplotypes are present in 15% and in 56% of the Wichí population respectively and differ for only one mutation in position 16422. More in details, as shown in Figure 11, mtHt4 is ancestral respect to the mtHt15.

Discrepancy was observed even between genealogical parental relationships based on self-reported identities and the NRY results both for SNPs and STRs among Wichí. Very different STRs haplotypes patterns brought us to consider that a particular idea of the family structure is common and that this has to be taken into account in the choice of unrelated samples. From the paternal perspective we obtained genetic data for 47 father/son, brothers and male cousins pairs. For these cases we found an agreement between self-reported kinship and genetic data. It is worth noticing that in nine haplotypes one or two loci are polymorphic in the time span of one generation. More in detail, the most variable locus is DYS439 (five changes) followed by DYS458 (three changes) and DYS393 with two changes, whereas GATA H4.1 and DYS391 change just once during generations. Furthermore, in three cases, haplotypes differ by two loci from the related haplotype and in one pair a mutation involves two changes (gain of repetition) in the same locus. These facts highlight the possibility of divergence among lineages in a very short time span, jumping from ancestral to derived haplotype. These very rare occurrences are previously reported by [31]. Conversely, a social explanation is provided by the extramarital relationships although this practice, as in the most of the human societies, has not a social establishment. In fact the correspondence between the cultural parental relationships and the genetic-genealogic trees is a topic in the choice of unrelated samples in the human genetic population studies, because the mating practices are often heterogeneous in the different ethnic groups. Therefore in Wichí we unraveled that 29 samples related according to the self-reported identity are actually characterized by so many mutations in the STRs loci to consider them unrelated samples. A plausible explanation lies in a particular manner to conceive the family structure in Wichí traditions. Indeed in the identification of relationships, the age of individuals more than real consanguinity plays an essential role [12].

Moreover, if, considering the maternal perspective, the reported 20% of Criollos individuals with Amerindian NRY have a Wichí ancestry (data not shown), and a comparable amount of Wichí has an European NRY, we can confirm the presence of bidirectional flows.

## Discussion

In genetic studies associations between genotypes and phenotypes may be confounded by unrecognized population structure and/or admixture. Genetic ancestry in recently admixed populations varies largely among individuals particularly when this variation is the result of the combination of genetic and/or cultural factors although self-reported population ancestry likely provides a suitable proxy for many applications in epidemiology, as well as for assessing disease risks [32]. In this study we typed 522 individuals from 2 neighboring populations from the Chaco regions in Argentina: Wichí, a Native American group, and Criollos, an admixed group, both sharing the same land and social environment.

The individuals, whose Wichí or Criollos ethnicity was preliminarily self-reported, were analyzed for mitochondrial genetic variability and 243 males for NRY diversity. On the basis of the Y-STRs haplotype relatedness and the genealogical and demographic reconstruction, performed according to the social relationships and the mating traditions, we identified 184 unrelated males in order to carry out a reliable survey, comparable with literature data. These individuals constituted the bulk of unrelated samples even for mitochondrial analyses. Nevertheless, analyses for both NRY and mtDNA carried out on the total samples, including closely related individuals on the basis of self-reported identity, gave us a unique perspective to dissect the complex kinship patterns of the families of the two sympatric groups.

The deep knowledge of the family structures, the mating dynamics and the genealogical trees, carried out during a long time fieldwork, revealed among Wichí some traditional complex kinship patterns that override the real genealogical definition. Some practices, as sororal polygyny (Text S3), post-marriage uxorilocal residence, bilateral descent or variability between endogamy and exogamy, produce an intricate pattern of relationships in the community, and deeply change the self-reported identity. For Wichí these well accepted and widely spread rules are mechanisms of alliance, a way to maintain social stability in the extended political units that represent the universe of reference for the individual. Paradigmatic is the case of the family reported in Figure12 that contrasts with the saying “mater semper certa est” especially when, comparing the profile of the NRY of the two sons, it is consistent with that of the father (ID 400, Wichí). These results are in agreement with the cultural relationship of “hermanos de crianza” or ”milk brothers”, which represents a sort of adoption [33]. If during the sampling we had considered the self-reported relationships and identities alone, we would have sampled only one child out of nine. On the contrary, with the sampling of almost half of the children we were able to detect a higher amount of “hidden” genetic diversity, due to the social structure dynamics.

In order to disentangle this complex scenario and to reconstruct the genetic structure and the pattern of admixture of Misión Nueva Pompeya population, we elaborated an integrated cultural and molecular anthropological model that reconsidered the sampling strategy in the fieldwork of human population genetic studies. This approach allowed us to unravel in a genetic survey all the mismatches between cultural and biological relationships. In this context it was critical the comparison with available literature data, carried out by common sampling methods, both to detect intra population genetic diversity and phylogenetic/phylogeographic relationships. Even though we are aware that regarding to uniparental markers genetic estimates they reflect only a little fraction of any person's total genetic ancestry, but, escaping recombination, they are able to detect the ancestral and admixed components of the two communities, our multidisciplinary study allowed us to understand the correlation between cultural rules and actual mating patterns that shapes the gene pool of the population.

Therefore, according to the aims of the present study, we were able to reconstruct the genetic structure and the pattern of admixture, also leading to verify the correspondence between genealogical and genetic relationships. This integrated perspective had the power to validate each other the data, to check the meaning of the results and to link the gap that usually relies on a singular source of information.

Wichí and Criollos, that are culturally two different populations even sharing the dwelling space *(parajes),* are differentiated also from the genetic point of view. In fact, AMOVA computations support the structure of two distinct populations, with significant values of Fst, for both female and male perspective. Moreover, the admixture analysis revealed more than 90% Amerindian ancestry for Wichí, while in Criollos this value is about 25%.

### Paternal Perspective

NRY genetic variability was very low in the Wichí and higher in the Criollos population. In general, among Wichí a higher homogeneity within the Amerindian lineages with a strong founder effect is observed, in contrast to the higher heterogeneity showed among Criollos (Figures 3 and 4). This fact is supported by significant and high values of Fst for NRY and depends on the European contribution which is much higher in Criollos, where the ratio of European/Native parental contribution is complementary to the one of Wichí. A clear difference in the genetic structure of Wichí and Criollos emerged from the haplotype diversity essentially resulting from the haplogroup diversification (Europeans versus Amerindian), as we can see in Figure 4 and 5. In such a way, the lower diversity observed in Wichí can be explained by the scarceness of European haplogroups.

The higher frequency of the Amerindian M3-Q1a3a lineage among Criollos in comparison with previously studied admixed populations suggests a significant gene flow from the Natives to the immigrants among Criollos, and an opposite smaller flow towards the Wichí group. Cultural traditions usually state that the flow is unidirectional, namely a mixed mating always generates a Criollo descendent, never a Wichí. Actually, other mechanisms preserve bidirectional flows, although preferably the Native/Criollo offspring adopt the status of Criollo, rather than Native Wichí.

On the other hand, European lineages characterize Criollos giving a wide spectrum of variability. From historical sources, two European waves arrived in Argentina. The first one essentially came from Spain during the post colonial age, the second one in the 19^th^ and 20^th^ centuries from all over Europe but especially from Italy and Spain (34% and 22% respectively of total immigrants according to [30], [34]). The resulting admixture components (using data from literature as parental haplotypes) revealed that the Spaniards were the most important presence in Gran Chaco colonizers peopling, followed by the Italian one (about 51% and 26% respectively).

After disentangling the Amerindian component from the Old World the phylogenetic relationships showed that the Gran Chaco gene pool from Misión Nueva Pompeya is rather structured and differentiated in the South American scenario (Figures 5 and 6). Wichí and Criollos cluster indipendently, sharing very few haplotypes with other Amerindian populations, except from Toba. The latter, mainly from the eastern Gran Chaco area, shares many lineages with Wichí and it seems to be highly related to Wichí ethnicities, despite their different traditions and cultures. Another important remark concerns the fact that the Criollos native component derives essentially from Wichí gene pool, as shown in the branches of the network departing from modal Wichí haplotype (Figure 3).

The European phylogenetic reconstruction on unrelated samples did not highlight an evidence of linkage with specific populations or lineages. In fact the variability of each haplogroup set up a homogenous cluster in which there are all populations, without any particular population structure. But taking the population as a whole, the European component of Wichí and Criollos fits within the Western Mediterranean cluster, together to Iberian and Italian samples.

### Maternal Perspective

Mismatch distribution assumed opposite trends in Wichí and Criollos with a multimodal distribution recognizable in Wichí stated a stationary population in the time, suggesting a more ancient settlement and adaptation to the territory. On the opposite, the unimodal-like trend resulted from Criollos haplotypes comparison, indicates a demographic expansion occurred in the past, probably since the Spanish conquest and colonization, in line with the historical events. Moreover, according to the expectation, genetic diversity (H) showed higher values for Criollos, increasing from the total to the unrelated sample, while in Wichí an opposite trend was shown. An explanation for this result is that the usual practice of reiterated marriages of a Wichí male with different females leads to an underestimation of the mtDNA variability when unrelated samples were considered.

Previous nucleotide diversity estimates in Gran Chaco region showed midrange values in comparison to higher values in Andean areas and lower in Tropical Forest [29]. Otherwise in Wichí and Criollos these parameters have extreme values in the range of π linking Wichí with Amazonian and Criollos with Andean populations.

The Multidimensional scaling representation of the genetic distances among a set of Amerindian populations from mtDNA haplotype estimates is remarkable in many aspects. The first image (Figure 9) reveals that Wichí and Criollos are in the same cluster of Andean and admixed populations respectively. On the contrary, plotting only unrelated samples, we obtained a very different pattern, in which Criollos cluster with Andean populations, but Wichí appear in the cluster of Amazonian populations (Figure 10). This unpredictable result shows the power of the correct choice of the samples which will represent the population, in contrast with some previous results on the Gran Chaco populations [29].

Finally, the MJN (Figure 11) on the phylogenetic relationships among the different haplotypes shows that Wichí and Criollos are characterized by several lineages not previously detected, with a peculiar structure within the Gran Chaco genetic landscape as previous studies already claimed. For this reason it will be interesting to sequence the whole mtDNA of these peculiar haplotypes. Moreover, except from haplogroup A4 and few samples in other haplogroups, Wichí and Criollos do not share haplotypes, despite all the lineages being Amerindian, supporting different ancestries and a scarceness of gene flow between them. In fact, more recent ancestries for C1 lineage, more ancient and with a strong founder effect for A2, sublineages peculiar for Wichí and for Criollos for both D1 and B4 are observed.

## Conclusions

Summarizing, Wichí and Criollos still preserve their genetic identity and peculiar characterization as a population, despite their sharing of the environment, of some *parajes* and even households. Nearly 20% is the quote of exchange for NRY between the two demes, in both directions, although the cultural practices suggest uni-directionality from Wichí to Criollos, and not the opposite. Phenotypic traits do not help in the distinction between the two populations. This fact generates a present day appearance of meta-population. Even the self identification often creates some mismatches with actual kinship, due to the local mating practices and social rules, deeply investigated in this work by means of Cultural Anthropology tools. This new insight in approaching anthropological cases leads to new research opportunities: first of all, we can discern actual components in human populations’ genetic structure. Starting from this basic step, some consequences come up. The population relationships and the reconstruction of past migrations as well as the investigation of different ancestries which originated current variability, are now much more reliable and close to the reality, avoiding bias caused by the relatedness among the samples. In such a way, clusterization and phylogenetic relationships in networks, MDS or other statistical methods suitable to represent the spatial distribution of the genetic distances or affinities, fit better with the reality.

In conclusion, we can affirm that anthropological genetics and cultural anthropology do not overlap but are complementary. The temporal diachronicity and synchronicity do not share the same pattern but are integrated; the populations are structured and differentiated, both from genetic and cultural points of view. Actual knowledge of mating dynamics sheds new insights in the population genetics results. These results state a very strong founder effect in Wichí lineages and a differentiated origin for Wichí and Criollos Amerindian component.

The pattern of admixture and the dissection of stratified populations will enable to apply a population-disease approach to test the hypothesis of a different genetic susceptibility to Chagas disease and its clinical outcomes, starting from the comparison between the distribution of the Chagas seroprevalence and the genetic lineages [13]. In fact this kind of study could have a very important effect on the evolutionary medicine sphere. Moreover this innovative branch that is getting more and more credit in the scientific community, must take into account the different ancestral components in the human diversification of the responses to the diseases and anti-inflammatory pathways. However we acknowledge that the uniparental markers used for this study can only be used at a population level. In order to better assess the mismatch between expected and observed level of admixture at an individual level, the analyses of autosomal markers will be needed. As the level of single individuals, only autosomic markers can indeed translate the presence/absence of European uniparental types into a proportion of genomic mixture. In Misión Nueva Pompeya region, the Chagas disease has very high rates of incidence, and the previous study [13] demonstrated that there are not significant differences between Wichí and Criollos affliction by disease, but these findings are based on self reported ethnicity affiliation whereas our methods provide the actual stratification, useful to perform in the future reliable comparative studies on susceptibility data between cases and controls taking into account the real subdivision of the two groups underlined by uniparental markers, meaning different pattern of neutral variants that could reflect other polymorphisms involved in more important metabolic pathways in the inflammatory stages.

## Supporting Information

Figure S1 Θ**_H_ values for Wichí and Criollos at each NRY locus.**
(TIF)

Figure S2 **Median Joining Network performed on 12 NRY loci haplotypes from Wichí and Criollos (in yellow and red respectively) and other Amerindian populations available in literature.**
(TIF)

Table S1 **Target sequences for NRY variability assessment.**
(DOC)

Table S2 **Amplification primers for NRY variability assessment.**
(DOC)

Table S3 **Populations used in NRY STRs haplotypes comparison.**
(DOC)

Table S4 **NRY haplotypes in the two populations**. (loci are in the following order: DYS19, DYS389I, DYS389II, DYS390, DYS391, DYS392, DYS393, DYS385a, DYS385b, DYS437, DYS438, DYS439, DYS448, DYS456, DYS458, DYS635, GATA H4).
(DOC)

Table S5 **Populations used for NRY haplogroups frequencies comparison and the relative references.**
(DOC)

Table S6 **mtDNA haplotypes, from 1 to 53, in the two populations.**
(DOC)

Table S7 **mtDNA haplotypes, from 54 to 106, in the two populations.**
(DOC)

Table S8 **Main genetic diversity parameters in Wichi and Criollos for mitochondrial D-loop sequences comparing unrelated and total samples.**
(DOC)

Table S9 **mtDNA sequences publicly available (only one per sub-haplogroup) included in MJ Network analysis (** **Figure 11** **).**
(DOC)

Text S1 **Methods for NRY variability assessment.**
(DOC)

Text S2 **Methods for mtDNA Haplogroup assignation.**
(DOC)

Text S3 **Sororate definition.**
(DOC)
