# Correction: Analysis of Population Substructure in Two Sympatric Populations of Gran Chaco, Argentina

**DOI:** 10.1371/annotation/623e9573-9757-42ba-96c3-870881faf06a

**Published:** 2014-01-03

**Authors:** Federica Sevini, Daniele Yang Yao, Laura Lomartire, Annalaura Barbieri, Dario Vianello, Gianmarco Ferri, Edgardo Moretti, Maria Cristina Dasso, Paolo Garagnani, Davide Pettener, Claudio Franceschi, Donata Luiselli, Zelda Alice Franceschi

There were errors in Supporting Tables S6 and S7.

Please see the corrected Table S6 here: 

Please see the corrected Table S7 here: 
