# Supplementary figures and images for: Analysis of Population Substructure in Two Sympatric Populations of Gran Chaco, Argentina
Source: PLoS One. 2013 May 22;8(5):e64054. doi: 10.1371/journal.pone.0064054 (PMC3661677; doi:10.1371/journal.pone.0064054)

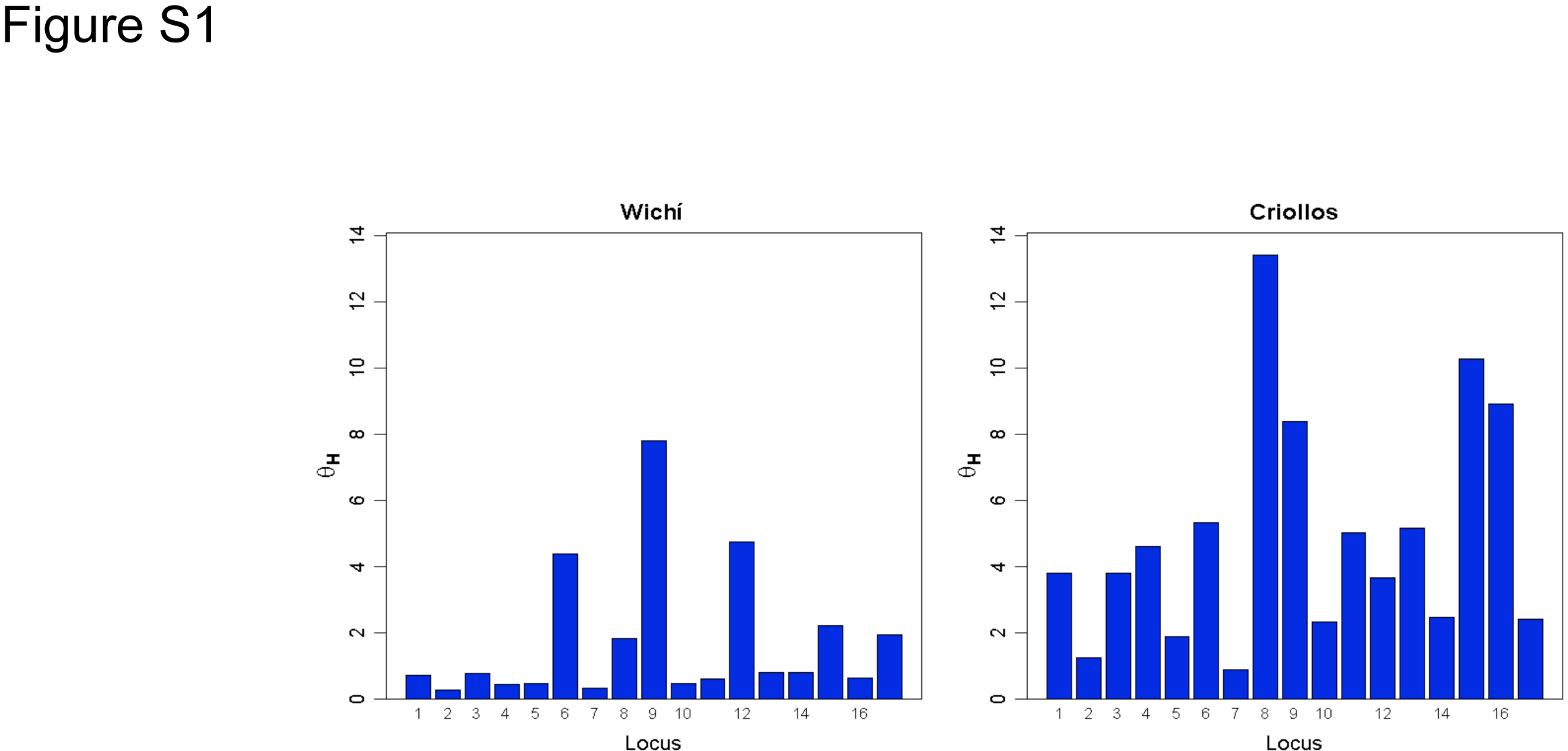

Supplement: Figure S1 — ΘH values for Wichí and Criollos at each NRY locus. (TIF) [file pone.0064054.s001.tif]

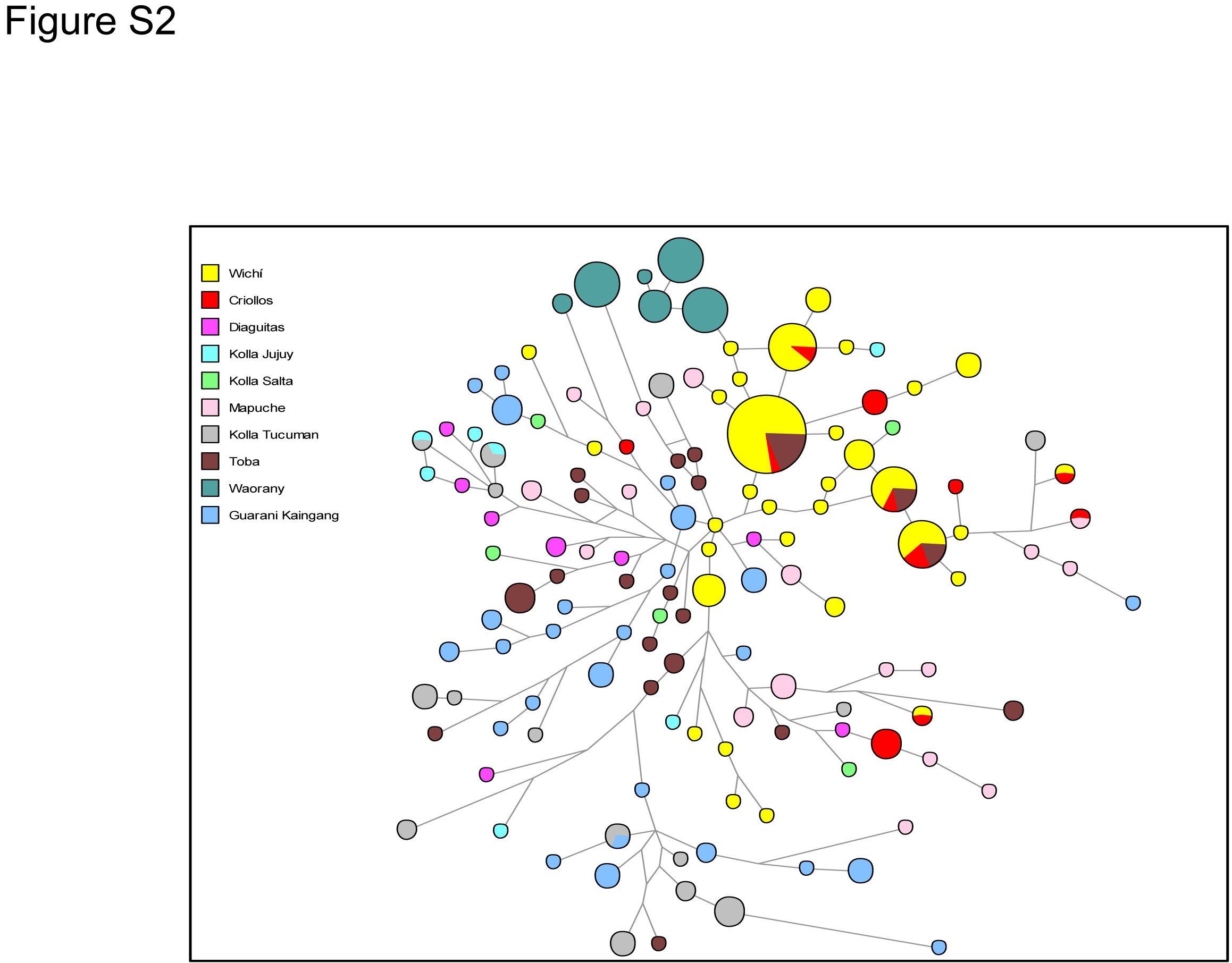

Supplement: Figure S2 — Median Joining Network performed on 12 NRY loci haplotypes from Wichí and Criollos (in yellow and red respectively) and other Amerindian populations available in literature. (TIF) [file pone.0064054.s002.tif]
