# Supplementary material for: Analysis of Population Substructure in Two Sympatric Populations of Gran Chaco, Argentina
Source: PLoS One. 2013 May 22;8(5):e64054. doi: 10.1371/journal.pone.0064054 (PMC3661677; doi:10.1371/journal.pone.0064054)
Supplement: Table S1 — Target sequences for NRY variability assessment. (DOC) [file pone.0064054.s003.doc]

**Table S1.** Target sequences for NRY variability assessment.

| **Sequence Label** | | **Sequence specific** | **Total length** | **Specific length** | **MUT** |
| --- | --- | --- | --- | --- | --- |
| **M242-R** | **ttt**AAACACGTTAAGACCAATGCCAA | AAACACGTTAAGACCAATGCCAA | 26 | 23 | **C/T** |
| **M194-R** | **(gact)_2_**AGTCGTTGCCTTCTCGGGGGGAA | AGTCGTTGCCTTCTCGGGGGGAA | 31 | 23 | **T/C** |
| **M199-F** | **(gact)_4_**TGTTAAAATGGCTTACACTTG | TGTTAAAATGGCTTACACTTG | 37 | 21 | **C/G** |
| **MEH2-R** | **(gact)_6_**AAAACTGCATTGATGAATTTTCT | AAAACTGCATTGATGAATTTTCT | 47 | 23 | **G/T** |
| **P36.2-R** | **(gact)_6_**ATCATCTATCTATCCATTATTCTCTCT | ATCATCTATCTATCCATTATTCTCTCT | 51 | 27 | **G/A** |
| **M346-F** | **(gact)_5_**GCAGCCAAGAGGACAGTAAGA | GCAGCCAAGAGGACAGTAAGA | 41 | 21 | **C/G** |
| **M19-F** | **(gact)_9_**ATTTTTGTGAAGACTGTTGTA | ATTTTTGTGAAGACTGTTGTA | 57 | 21 | **T/A** |
| **M3-R** | AGGTACCAGCTCTTCCTAATT | AGGTACCAGCTCTTCCTAATT | 21 | 21 | **C/T** |
