# Supplementary material for: Analysis of Population Substructure in Two Sympatric Populations of Gran Chaco, Argentina
Source: PLoS One. 2013 May 22;8(5):e64054. doi: 10.1371/journal.pone.0064054 (PMC3661677; doi:10.1371/journal.pone.0064054)
Supplement: Table S2 — Amplification primers for NRY variability assessment. (DOC) [file pone.0064054.s004.doc]

**Table S2.** Amplification primers for NRY variability assessment.

| **Marker** | **Primer (5'-3')** |  | **Amplicon size** | **Mut** |
| --- | --- | --- | --- | --- |
|  | **Forward** | **Reverse** |  |  |
| **M242** | GTGCAAAAAGGTGACCAAGG | GCTTTAAGGGCTTTCAGCAT | **149** | **C/T** |
| **M194** | TGGATGAGGAAGTGAGTCCTG | TTATACAGTCGTTGCCTTCTCG | **126** | **T/C** |
| **M199** | TTGTGCAGCAGGCTTTAATTT | TGATTTCAAGGATTTGTTAGTCTT | **122/123** | **insG** |
| **MEH2** | GAGTAAGCCATCACCCCAAT | TGCAAAAACTGCATTGATGA | **80** | **G/T** |
| **P36.2** | AGGAGGGGGAGAGAGAAAAAG | CATCCATCCATGAACTGCTTC | **229** | **G/A** |
| **M346** | TCCCTCTATCCTCGATGCTTT | TCCACTCACTCTGCCTACCTG | **144** | **C/G** |
| **M19** | GGGTTTGTTAAGTGGCCAAA | GCTGACCACAAACTGATGTAGAG | **82** | **T/A** |
| **M3** | AATGTGGCCAAGTTTTATCTGC | TTGTGAATCTGAAATTTAAGGGC | **131** | **C/T** |
