# Supplementary material for: Analysis of Population Substructure in Two Sympatric Populations of Gran Chaco, Argentina
Source: PLoS One. 2013 May 22;8(5):e64054. doi: 10.1371/journal.pone.0064054 (PMC3661677; doi:10.1371/journal.pone.0064054)
Supplement: Table S3 — Populations used in NRY STRs haplotypes comparison. (DOC) [file pone.0064054.s005.doc]

**Table S3.** Populations used in NRY STRs haplotypes comparison.

| **Populations** | **Country** | **Reference** | **N (3147)** |
| --- | --- | --- | --- |
| Diaguitas | Argentina | Blanco Verea et al., 2010 | 9 |
| Kolla | Argentina | Blanco Verea et al., 2010 | 12 |
| Mapuche | Argentina | Blanco Verea et al., 2010 | 23 |
| Kolla | Argentina | Toscanini et al., 2008 | 29 |
| Toba | Argentina | Toscanini et al., 2008 | 31 |
| Waorany | Ecuador | Gonzalez-Andrade et al., 2009 | 35 |
| Quechua | Ecuador | Gonzalez-Andrade et al., 2007 | 102 |
| Quito | Ecuador | Baeza et al., 2007 | 120 |
| Peru | Peru | Iannacone et al., 2005 | 79 |
| Guarani and Kaingang | Brazil | Leite et al., 2008 | 42 |
| Rio Grande do Sul | Brazil | Leite et al.,2008 | 203 |
| Sao Paolo | Brazil | De Souza et al., 2005 | 126 |
| African Rio de Janeiro | Brazil | Domingues et al., 2007 | 135 |
| Sao Paolo | Brazil | Gois et al., 2007 | 200 |
| Rio de Janeiro | Brazil | Pena et al., 2006 | 120 |
| Colombia | Colombia | Romero et al., 2008 | 305 |
| South West Colombia | Colombia | Gomez et al., 2008 | 308 |
| Cartagena | Colombia | Builes et al., 2006 | 173 |
| Culis | Costa Rica | Castrì et al., 2007 | 25 |
| North Portugal | Portugal | Pontes et al., 2007 | 175 |
| Portugal | Portugal | Alves et al., 2007 | 250 |
| North East Italy | Italy | Turrina et al., 2006 | 155 |
| Modena | Italy | Ferri et al., 2008 | 130 |
| Marche | Italy | Onori et al., 2007 | 162 |
| Spain | Spain | Martin et al., 2004 | 148 |
| South Iberia | Spain | Gaibar et al., 2010 | 50 |
