# Supplementary material for: Analysis of Population Substructure in Two Sympatric Populations of Gran Chaco, Argentina
Source: PLoS One. 2013 May 22;8(5):e64054. doi: 10.1371/journal.pone.0064054 (PMC3661677; doi:10.1371/journal.pone.0064054)
Supplement: Table S4 — NRY haplotypes in the two populations. (loci are in the following order: DYS19, DYS389I, DYS389II, DYS390, DYS391, DYS392, DYS393, DYS385a, DYS385b, DYS437, DYS438, DYS439, DYS448, DYS456, DYS458, DYS635, GATA H4). (DOC) [file pone.0064054.s006.doc]

**Table S4.** NRY haplotypes in the two populations (loci are in the following order: DYS19, DYS389I, DYS389II, DYS390, DYS391, DYS392, DYS393, DYS385a, DYS385b, DYS437, DYS438, DYS439, DYS448, DYS456, DYS458, DYS635, GATA H4).

| ID | Total  (184) | Wichi (n=100) | Criollos  (n=84) | Haplotype | HG |
| --- | --- | --- | --- | --- | --- |
| CW1 | 3 | 3 | 0 | 13 13 31 24 10 14 13 13 17 14 11 12 21 15 17 22 12 | Q1a3a* |
| CW2 | 7 | 6 | 1 | 13 13 30 24 10 15 13 15 16 14 11 14 20 15 17 22 12 | Q1a3a* |
| CW3 | 18 | 17 | 1 | 13 13 30 24 10 15 13 15 16 14 11 13 20 15 17 22 12 | Q1a3a* |
| CW4 | 2 | 2 | 0 | 13 13 30 24 10 16 13 15 17 14 11 13 20 16 16 22 11 | Q1a3a* |
| CW5 | 5 | 3 | 2 | 14 13 30 22 9 12 12 13 14 15 9 12 20 15 17 24 11 | J2 |
| CW6 | 4 | 4 | 0 | 13 13 30 24 10 14 13 15 21 14 11 13 20 15 18 22 11 | Q1a3a* |
| CW7 | 6 | 5 | 1 | 13 13 30 24 10 14 13 15 21 14 11 12 20 15 18 22 11 | Q1a3a* |
| CW8 | 1 | 1 | 0 | 14 13 29 25 10 14 13 11 14 15 12 11 22 15 15 24 12 | R1 |
| CW9 | 1 | 1 | 0 | 15 12 29 22 11 11 14 14 14 15 10 10 21 14 17 21 12 | G |
| CW10 | 1 | 1 | 0 | 13 13 30 24 10 16 13 16 17 14 11 13 20 16 16 22 11 | Q1a3a* |
| CW11 | 2 | 2 | 0 | 13 13 30 24 10 15 13 15 16 14 11 14 20 15 18 22 12 | Q1a3a* |
| CW12 | 1 | 1 | 0 | 13 13 30 24 10 14 13 14 17 14 11 12 20 16 17 22 12 | Q1a3a* |
| CW13 | 2 | 2 | 0 | 13 13 29 24 10 14 13 14 17 14 11 11 20 16 17 22 13 | Q1a3a* |
| CW14 | 1 | 1 | 0 | 13 13 31 24 10 14 13 13 17 14 11 12 21 15 17 22 11 | Q1a3a* |
| CW15 | 1 | 1 | 0 | 13 13 30 24 10 15 13 15 16 14 11 13 20 15 16 22 10 | Q1a3a* |
| CW16 | 1 | 1 | 0 | 13 13 30 24 10 15 13 15 16 14 11 14 20 15 17 22 11 | Q1a3a* |
| CW17 | 1 | 1 | 0 | 13 13 30 24 10 16 13 15 17 14 11 13 20 16 16 22 9 | Q1a3a* |
| CW18 | 1 | 1 | 0 | 13 13 30 24 10 14 13 15 20 14 11 12 20 15 18 22 9 | Q1a3a* |
| CW19 | 1 | 1 | 0 | 13 13 30 24 10 14 13 15 21 14 11 12 20 15 18 22 10 | Q1a3a* |
| CW20 | 1 | 1 | 0 | 13 13 31 24 10 15 13 15 16 14 11 13 20 15 17 22 11 | Q1a3a* |
| CW21 | 1 | 1 | 0 | 13 13 30 24 10 15 13 15 16 14 11 15 20 15 17 22 11 | Q1a3a* |
| CW22 | 1 | 1 | 0 | 13 12 29 24 10 15 13 15 16 14 11 13 20 15 16 22 10 | Q1a3a* |
| CW23 | 4 | 4 | 0 | 14 14 30 24 11 13 13 11 13 15 13 12 19 16 17 24 12 | R1 |
| CW24 | 1 | 1 | 0 | 14 13 30 24 10 15 13 15 16 14 11 14 20 15 17 22 12 | Q1a3a* |
| CW25 | 1 | 1 | 0 | 13 13 30 24 10 14 13 13 17 14 11 12 21 15 17 22 12 | Q1a3a* |
| CW26 | 1 | 1 | 0 | 14 13 30 24 11 13 13 11 14 14 12 12 18 16 17 24 12 | R1 |
| CW27 | 2 | 2 | 0 | 13 13 30 24 10 15 13 15 16 14 11 15 20 15 17 22 12 | Q1a3a* |
| CW28 | 6 | 1 | 5 | 14 13 29 25 11 13 13 11 14 15 12 12 18 15 17 24 12 | R1 |
| CW29 | 1 | 1 | 0 | 13 13 30 24 11 15 13 15 16 14 11 14 20 15 17 22 12 | Q1a3a* |
| CW30 | 1 | 1 | 0 | 13 13 31 24 10 14 13 15 21 14 11 12 20 15 18 22 11 | Q1a3a* |
| CW31 | 2 | 1 | 1 | 13 13 29 24 9 14 13 13 14 15 11 11 20 16 15 22 11 | Q1a3a* |
| CW32 | 1 | 1 | 0 | 13 11 28 23 11 14 12 15 17 14 11 12 19 15 14 22 12 | Q1a3a* |
| CW33 | 1 | 1 | 0 | 13 13 31 24 10 14 13 15 21 14 11 13 20 15 18 22 11 | Q1a3a* |
| CW34 | 4 | 3 | 1 | 13 13 30 24 10 14 13 15 20 14 11 12 20 15 18 22 11 | Q1a3a* |
| CW35 | 3 | 2 | 1 | 13 13 30 24 10 14 13 15 20 14 11 12 20 16 18 22 11 | Q1a3a* |
| CW36 | 1 | 1 | 0 | 13 13 30 24 10 15 13 15 16 14 11 13 20 15 18 22 12 | Q1a3a* |
| CW37 | 1 | 1 | 0 | 13 13 30 25 10 15 13 15 16 14 11 13 20 15 17 22 12 | Q1a3a* |
| CW38 | 1 | 1 | 0 | 13 13 30 24 10 14 13 15 19 14 11 12 20 15 17 22 11 | Q1a3a* |
| CW39 | 1 | 1 | 0 | 13 13 30 24 10 14 13 14 14 14 11 13 20 16 18 18 12 | Q1a3a* |
| CW40 | 1 | 1 | 0 | 14 13 30 24 10 14 13 15 20 14 11 12 20 15 18 22 11 | Q1a3a* |
| CW41 | 1 | 1 | 0 | 13 13 30 24 10 15 11 15 16 14 11 12 20 15 17 22 12 | Q1a3a* |
| CW42 | 1 | 1 | 0 | 13 13 30 24 10 15 13 15 16 14 11 12 20 15 17 22 12 | Q1a3a* |
| CW43 | 1 | 1 | 0 | 14 14 30 24 11 13 13 11 13 15 13 12 19 15 17 24 12 | R1 |
| CW44 | 1 | 1 | 0 | 13 13 31 24 10 14 11 13 17 14 11 12 21 15 17 22 12 | Q1a3a* |
| CW45 | 1 | 1 | 0 | 13 13 30 23 10 14 11 14 14 14 11 13 20 16 18 22 12 | Q1a3a* |
| CW46 | 1 | 1 | 0 | 13 13 30 24 10 14 13 15 20 14 11 11 20 16 18 22 11 | Q1a3a* |
| CW47 | 1 | 1 | 0 | 13 13 30 24 10 15 11 15 16 14 11 15 20 15 17 22 12 | Q1a3a* |
| CW48 | 1 | 1 | 0 | 14 13 30 24 10 15 13 15 16 14 11 13 20 15 17 22 12 | Q1a3a* |
| CW49 | 1 | 1 | 0 | 13 13 30 24 10 15 13 15 16 14 11 13 20 17 17 22 12 | Q1a3a* |
| CW50 | 1 | 1 | 0 | 14 13 29 25 10 13 13 11 14 15 12 12 18 15 17 22 12 | R1 |
| CW51 | 1 | 1 | 0 | 13 13 31 24 10 11 13 17 17 14 10 12 20 16 15 23 11 | E1b1b1 |
| CW52 | 1 | 1 | 0 | 13 13 31 24 10 14 13 13 17 14 11 12 21 15 16 22 12 | Q1a3a* |
| CW53 | 1 | 1 | 0 | 14 13 30 23 10 11 12 13 16 14 10 13 21 15 17 21 11 | J1 |
| CW54 | 2 | 1 | 1 | 15 13 30 23 10 15 13 16 17 15 11 12 19 16 18 22 11 | Q1a3a* |
| CW55 | 2 | 1 | 1 | 15 13 31 23 11 11 13 11 14 14 11 10 18 16 14 23 12 | R1 |
| CW56 | 1 | 1 | 0 | 14 13 29 25 10 13 13 11 14 15 12 12 18 15 17 23 12 | R1 |
| CW57 | 1 | 1 | 0 | 14 14 30 23 10 14 12 16 17 16 10 11 20 14 16 21 10 | L |
| CW58 | 1 | 1 | 0 | 16 12 28 24 9 11 12 14 17 14 9 12 20 15 14 21 12 | J2 |
| CW59 | 1 | 0 | 1 | 14 13 31 23 10 11 12 13 18 14 10 11 20 15 17 21 11 | J1 |
| CW60 | 1 | 0 | 1 | 14 13 29 24 11 13 14 11 14 15 12 11 19 16 18 25 12 | R1 |
| CW61 | 1 | 0 | 1 | 13 14 30 24 9 11 13 13 14 14 10 10 20 16 18 21 12 | E1b1b1 |
| CW62 | 2 | 0 | 2 | 14 13 29 20 10 13 13 11 14 15 12 13 19 15 19 23 12 | R1 |
| CW63 | 1 | 0 | 1 | 14 13 29 24 10 13 13 11 14 15 12 12 20 17 16 23 12 | R1 |
| CW64 | 1 | 0 | 1 | 15 13 31 24 11 11 13 10 14 14 11 10 19 16 16 24 12 | R1 |
| CW65 | 1 | 0 | 1 | 13 13 31 24 10 11 13 17 18 15 10 12 19 15 17 23 11 | E1b1b1 |
| CW66 | 3 | 0 | 3 | 13 13 30 24 10 15 13 16 17 14 11 13 20 15 13 22 12 | Q1a3a* |
| CW67 | 1 | 0 | 1 | 15 12 29 24 10 10 12 15 17 14 10 12 20 15 15 22 11 | E1b1b1 |
| CW68 | 2 | 0 | 2 | 15 12 29 24 10 11 13 17 18 14 10 12 20 15 15 22 11 | E1b1b1 |
| CW69 | 1 | 0 | 1 | 13 13 29 23 10 11 13 14 16 14 10 12 20 17 14 21 11 | E1b1b1 |
| CW70 | 1 | 0 | 1 | 14 13 30 24 11 13 12 11 15 15 12 12 19 15 16 23 11 | R1 |
| CW71 | 1 | 0 | 1 | 15 13 29 24 11 13 13 11 15 15 12 13 19 14 18 23 13 | R1 |
| CW72 | 1 | 0 | 1 | 15 13 29 24 11 13 13 11 14 15 12 12 19 17 17 24 12 | R1 |
| CW73 | 2 | 0 | 2 | 13 13 30 23 10 14 13 15 19 14 11 11 19 15 16 23 13 | Q1a3a* |
| CW74 | 1 | 0 | 1 | 14 12 29 22 10 11 13 13 14 16 10 12 20 15 14 21 11 | I |
| CW75 | 1 | 0 | 1 | 13 13 29 24 10 11 12 10 16 15 9 11 21 14 15 21 12 | J2 |
| CW76 | 2 | 0 | 2 | 14 13 29 24 11 13 13 11 14 15 12 12 19 15 17 23 12 | R1 |
| CW77 | 4 | 0 | 4 | 14 13 31 24 11 13 13 11 14 14 12 12 18 15 17 24 11 | R1 |
| CW78 | 3 | 0 | 3 | 14 14 30 23 10 13 13 11 14 15 12 13 19 16 16 24 11 | R1 |
| CW79 | 1 | 0 | 1 | 14 13 29 25 11 13 13 11 11 15 12 12 19 15 17 23 13 | R1 |
| CW80 | 1 | 0 | 1 | 14 13 29 25 11 13 13 11 11 15 12 12 19 15 17 22 13 | R1 |
| CW81 | 2 | 0 | 2 | 15 12 29 22 10 11 14 14 16 16 10 11 21 15 15 20 11 | G |
| CW82 | 2 | 0 | 2 | 16 12 29 23 10 12 15 14 15 15 10 11 19 14 16 19 12 | I |
| CW83 | 2 | 0 | 2 | 14 13 30 23 11 11 12 13 19 14 10 10 20 14 18 22 11 | J1 |
| CW84 | 2 | 0 | 2 | 13 13 30 23 10 14 13 15 19 14 11 11 19 15 17 23 13 | Q1a3a* |
| CW85 | 2 | 0 | 2 | 14 12 28 24 10 13 13 10 14 14 12 12 18 15 16 23 11 | R1 |
| CW86 | 1 | 0 | 1 | 14 13 29 24 10 14 13 15 16 14 11 11 19 16 18 22 12 | Q1a3a* |
| CW87 | 1 | 0 | 1 | 14 13 29 24 11 13 13 12 15 15 12 13 18 16 18 23 12 | R1 |
| CW88 | 1 | 0 | 1 | 15 12 29 22 10 11 14 12 16 16 10 12 21 18 18 22 12 | G |
| CW89 | 1 | 0 | 1 | 15 14 31 24 10 14 13 16 17 15 11 10 19 15 16 22 12 | Q1a3a* |
| CW90 | 1 | 0 | 1 | 13 12 30 24 10 14 13 16 17 14 11 12 21 15 18 22 11 | Q1a3a* |
| CW91 | 1 | 0 | 1 | 15 12 29 22 10 11 14 12 16 16 10 12 21 18 18 22 13 | G |
| CW92 | 1 | 0 | 1 | 15 13 30 25 10 11 13 11 14 14 11 9 20 17 15 23 12 | R1 |
| CW93 | 1 | 0 | 1 | 15 12 29 22 10 11 14 12 16 16 10 12 21 18 18 22 11 | G |
| CW94 | 1 | 0 | 1 | 14 12 29 22 10 11 13 13 14 16 10 12 20 15 19 21 11 | I |
| CW95 | 1 | 0 | 1 | 14 13 30 24 11 13 13 11 14 14 12 14 18 16 17 24 12 | R1 |
| CW96 | 1 | 0 | 1 | 13 13 28 22 10 13 13 12 15 15 9 11 19 15 15 21 11 | T |
| CW97 | 1 | 0 | 1 | 14 13 29 25 11 13 13 11 14 15 12 12 19 16 17 23 11 | R1 |
| CW98 | 1 | 0 | 1 | 13 14 30 24 9 11 13 13 14 14 10 10 19 15 18 21 12 | E1b1b1 |
| CW99 | 1 | 0 | 1 | 13 14 30 24 9 11 13 13 15 14 10 10 20 16 17 21 12 | E1b1b1 |
| CW100 | 2 | 0 | 2 | 15 13 30 23 10 11 12 13 20 14 10 12 20 16 18 21 11 | J1 |
| CW101 | 1 | 0 | 1 | 14 13 28 25 10 13 13 11 14 15 12 11 19 16 16 23 12 | R1 |
| CW102 | 1 | 0 | 1 | 14 12 28 26 10 13 13 11 14 15 12 12 18 15 18 23 11 | R1 |
| CW103 | 1 | 0 | 1 | 16 13 30 25 11 11 13 14 15 15 10 12 19 16 18 22 11 | I |
| CW104 | 1 | 0 | 1 | 13 14 31 24 9 13 13 14 16 14 13 12 20 15 18 22 12 | R1 |
| CW105 | 1 | 0 | 1 | 14 14 30 24 11 13 13 11 14 15 12 12 19 16 16 23 11 | R1 |
| CW106 | 1 | 0 | 1 | 14 13 30 23 10 11 12 13 15 14 10 12 21 15 17 21 11 | J1 |
| CW107 | 1 | 0 | 1 | 13 13 31 24 10 11 13 16 17 14 10 12 20 16 15 23 11 | E1b1b1 |
| CW108 | 1 | 0 | 1 | 14 13 29 24 11 13 13 12 14 15 12 11 19 15 18 23 12 | R1 |
| CW109 | 1 | 0 | 1 | 15 13 29 23 10 13 12 14 17 15 12 11 19 15 17 23 11 | R1 |
| CW110 | 1 | 0 | 1 | 14 13 30 24 10 11 13 16 19 14 10 12 20 16 15 22 12 | E1b1b1 |
