# Supplementary material for: Analysis of Population Substructure in Two Sympatric Populations of Gran Chaco, Argentina
Source: PLoS One. 2013 May 22;8(5):e64054. doi: 10.1371/journal.pone.0064054 (PMC3661677; doi:10.1371/journal.pone.0064054)
Supplement: Table S5 — Populations used for NRY haplogroups frequencies comparison and the relative references. (DOC) [file pone.0064054.s007.doc]

**Table S5.** Populations used for NRY haplogroups frequencies comparison and the relative references.

| **Populations** | **Country** | **Reference** | **N (2151)** |
| --- | --- | --- | --- |
| Extended group | South America | Bortolini et al., 2003 | 390 |
| Guarani and Kaingang | Brazil | Marrero et al., 2007 | 136 |
| Culis | Costa Rica | Castrì et al., 2007 | 25 |
| Costa Rica and Panama | Costa and Panama | Ruiz Narvaez et al., 2005 | 93 |
| Gran Chaco | Argentina | Demarchi et al., 2004 | 60 |
| Quechua | Ecuador | Gonzalez-Andrade et al., 2007 | 102 |
| Extended group | South America | Mazieres et al., 2009 | 179 |
| *Amerindian* | | | *985* |
| Andalusian and Catalans | Spain | Bosch et al., 2001 | 53 |
| Asturia | Spain | Scozzari et al., 2001 | 90 |
| Galizia and Valencia | Spain | Brion et al., 2003 | 84 |
| Italy, Spain and Portugal | Italy, Spain and Portugal | Rosser et al., 2000 | 610 |
| Italy | Italy | Semino et al., 2000 | 87 |
| Cantabria | Spain | Maca-Meyer et al., 2003 | 242 |
| *Europe* | | | *1166* |
