# Supplementary material for: Analysis of Population Substructure in Two Sympatric Populations of Gran Chaco, Argentina
Source: PLoS One. 2013 May 22;8(5):e64054. doi: 10.1371/journal.pone.0064054 (PMC3661677; doi:10.1371/journal.pone.0064054)
Supplement: Table S6 — mtDNA haplotypes, from 1 to 53, in the two populations. (DOC) [file pone.0064054.s008.doc]

**Table S6.** mtDNA haplotypes, from 1 to 53, in the two populations.
