# Supplementary material for: Analysis of Population Substructure in Two Sympatric Populations of Gran Chaco, Argentina
Source: PLoS One. 2013 May 22;8(5):e64054. doi: 10.1371/journal.pone.0064054 (PMC3661677; doi:10.1371/journal.pone.0064054)
Supplement: Table S8 — Main genetic diversity parameters in Wichi and Criollos for mitochondrial D-loop sequences comparing unrelated and total samples. (DOC) [file pone.0064054.s010.doc]

**Table S8.** Main genetic diversity parameters in Wichi and Criollos for mitochondrial D-loop sequences comparing unrelated and total samples.

|  |  |  | **Mismatch distribution** | | | | **Neutrality tests** | |
| --- | --- | --- | --- | --- | --- | --- | --- | --- |
| **Population** |  |  | **Demographic** | | **Spatial** | |  |  |
|  |  | **π** | **HR-i** | **P** | **HR-i** | **P** | **D** | **P-v** |
| **Wichi Total** |  | 0.011910 +/- 0.006048 | 0.05215 | 0.02000 | 0.05215 | 0.49000 | 0.05551 | 0.61300 |
| **Criollos Total** |  | 0.015311 +/- 0.007673 | 0.00644 | 0.20000 | 0.00644 | 0.90000 | -1.01494 | 0.16000 |
| **Wichi** | **HG A4** | 0.000438 +/- 0.000475 | 0.73533 | 0.83000 | 0.73533 | 0.72000 | -2.29266 | 0.00000 |
|  | **HG B4** | 0.002882 +/- 0.001765 | 0.02939 | 0.99700 | 0.02939 | 0.66100 | -0.61952 | 0.28800 |
|  | **HG C1** | 0.007375 +/- 0.004224 | 0.07577 | 0.33500 | 0.07577 | 0.78500 | -0.06754 | 0.50800 |
|  | **HG D4** | 0.003292 +/- 0.001947 | 0.07537 | 1.00000 | 0.07537 | 0.79500 | -0.82172 | 0.23900 |
| **Criollos** | **HG A4** | 0.001982 +/- 0.001321 | 0.06434 | 0.79000 | 0.06434 | 0.87000 | -1.66436 | 0.03000 |
|  | **HG B4** | 0.008270 +/- 0.004421 | 0.04348 | 0.06000 | 0.04348 | 0.81800 | -1.23533 | 0.09700 |
|  | **HG C1** | 0.007010 +/- 0.003767 | 0.01569 | 0.43400 | 0.01569 | 0.94300 | -0.69408 | 0.27100 |
|  | **HG D4** | 0.007626 +/- 0.004102 | 0.09468 | 0.01700 | 0.09468 | 0.36400 | 0.20532 | 0.64300 |
| **Wichi Unrelated** |  | 0.011679 +/- 0.005984 | 0.07684 | 0.00100 | 0.07684 | 0.42500 | 0.79258 | 0.83000 |
| **Criollos Unrelated** |  | 0.015358 +/- 0.007759 | 0.00341 | 0.81000 | 0.00341 | 0.99100 | -0.98700 | 0.15800 |
| **Wichi** | **U_HG A4** | 0.000900 +/- 0.000763 | 0.78331 | 0.76500 | 0.78331 | 0.74200 | -2.05169 | 0.00500 |
|  | **U_HG B4** | 0.002313 +/- 0.001552 | 0.17069 | 0.06700 | 0.17069 | 0.10800 | -0.70139 | 0.25500 |
|  | **U_HG C1** | - | - | - | - | - | - | - |
|  | **U_HG D4** | 0.004347 +/- 0.002495 | 0.10881 | 0.38900 | 0.10881 | 0.61500 | -1.20812 | 0.10900 |
| **Criollos** | **U_HG A4** | 0.002927 +/- 0.001879 | 0.06528 | 0.90700 | 0.06528 | 0.56200 | -1.04283 | 0.17700 |
|  | **U_HG B4** | 0.009131 +/- 0.005003 | 0.01628 | 0.75700 | 0.01628 | 0.95800 | -0.76295 | 0.25100 |
|  | **U_HG C1** | 0.006167 +/- 0.003482 | 0.02791 | 0.49500 | 0.02791 | 0.66300 | -1.36108 | 0.07800 |
|  | **U_HG D4** | 0.008715 +/- 0.004779 | 0.07315 | 0.11600 | 0.07315 | 0.42000 | 0.53270 | 0.74100 |

π: nucleotide diversity; HR-i: Harpending's Raggedness index; D: Tajima’s Neutrality index; Fu: Fu’s Neutrality index.
