# Supplementary material for: Analysis of Population Substructure in Two Sympatric Populations of Gran Chaco, Argentina
Source: PLoS One. 2013 May 22;8(5):e64054. doi: 10.1371/journal.pone.0064054 (PMC3661677; doi:10.1371/journal.pone.0064054)
Supplement: Table S9 — mtDNA sequences publicly available (only one per sub-haplogroup) included in MJ Network analysis ( Figure 11 ). (DOC) [file pone.0064054.s011.doc]

**Table S9.** mtDNA sequences publicly available (only one per sub-haplogroup) included in MJ Network analysis (Figure 11).

|  | **Accession number** | **Country** | **Haplogroup** | **Reference** |
| --- | --- | --- | --- | --- |
|  | ABS138 | Argentina | B2b | Bobillo et al. 2009 |
|  | HM107355 | Argentina | C1d1 | Perego et al. 2010 |
|  | ABS163 | Argentina | C1b6 | Bobillo et al. 2009 |
|  | HM107316 | Argentina | C1d | Perego et al. 2010 |
|  | ABS175 | Argentina | A2l | Bobillo et al. 2009 |
|  | ABS194 | Argentina | D4h3a | Bobillo et al. 2009 |
|  | ABS215 | Argentina | A2m | Bobillo et al. 2009 |
|  | HM107327 | Argentina | C1d1b1 | Perego et al. 2010 |
|  | ABS239 | Argentina | D1f | Bobillo et al. 2009 |
|  | HM107346 | Argentina | C1d1d | Perego et al. 2010 |
|  | ACA359 | Argentina | C1b | Bobillo et al. 2009 |
|  | ACH363 | Argentina | D1e1 | Bobillo et al. 2009 |
|  | AMI029 | Argentina | A2 | Bobillo et al. 2009 |
|  | AMI036 | Argentina | D1 | Bobillo et al. 2009 |
| AMI044 | | Argentina | B2 | Bobillo et al. 2009 |
| AMI046 | | Argentina | C1c | Bobillo et al. 2009 |
| ARN083 | | Argentina | B2e1 | Bobillo et al. 2009 |
|  | ARN109 | Argentina | B2e2 | Bobillo et al. 2009 |
|  | HM107351 | Argentina | C1d1e | Perego et al. 2010 |
|  | DQ282474 | USA | C1d1c1 | Achilli et al. 2008 |
|  | HM107314 | Colombia | C1d2a | Perego et al. 2010 |
|  | HM107321 | Mexico | C1d1a1 | Perego et al. 2010 |
|  | HM107323 | Argentina | C1d1b | Perego et al. 2010 |
|  | LPAZ001 | Bolivia | B4 | Afonso Costa et al. 2010 |
|  | LPAZ002 | Bolivia | D1 | Afonso Costa et al. 2010 |
|  | LPAZ050 | Bolivia | B4c1a | Afonso Costa et al. 2010 |
|  | LPAZ058 | Bolivia | D4j | Afonso Costa et al. 2010 |
|  | LPAZ059 | Bolivia | A4 | Afonso Costa et al. 2010 |
|  | Fag05_SURU01 | Brazil | A2 | Fagundes et al. 2008 |
|  | Fag14_GAVI23 | Brazil | B2 | Fagundes et al. 2008 |
|  | Fag29_WAI16 | Brazil | C1 | Fagundes et al. 2008 |
|  | FJ168712 | China | D4h3b | Perego et al. 2010 |
|  | FJ168719 | Chile | D4h3a1 | Perego et al. 2010 |
|  | FJ168726 | Chile | D4h3a2 | Perego et al. 2010 |
|  | FJ168731 | Mexico | D4h3a3 | Perego et al. 2010 |
|  | FJ168736 | Peru | D4h3a4 | Perego et al. 2010 |
|  | FJ168741 | Chile | D4h3a5 | Perego et al. 2010 |
|  | FJ168743 | Peru | D4h3a | Perego et al. 2010 |
