# Supplementary material for: Analysis of Population Substructure in Two Sympatric Populations of Gran Chaco, Argentina
Source: PLoS One. 2013 May 22;8(5):e64054. doi: 10.1371/journal.pone.0064054 (PMC3661677; doi:10.1371/journal.pone.0064054)
Supplement: Text S1 — Methods for NRY variability assessment. (DOC) [file pone.0064054.s012.doc]

**Text S1 Methods for NRY variability assessment**

All the biallelic markers (SNPs and STRs) were typed in two different multiplexes by using QIAGEN® Multiplex PCR kit and the SNaPshot™ Multiplex kit (Applied Biosystems). The first multiplex allowed to identify basal haplogroups defining main continent specific lineages. All primers and PCR conditions are described in Brion et al., (2004). Second multiplex aimed to explore intra-lineage diversity in Q haplogroup, diagnostic and very informative of Amerindian ancestry. For this we created a novel multiplex hierarchically typed only in M3 samples. Amplification primers design are reported in Tables S1 and S2. PCR conditions are the same above mentioned.

17 STRs markers were genotyped with AmpFlSTR® Yfiler™ multiplex kit (Applied Biosystems) following manufacturer recommendations for PCR conditions.

Both the Y-STR and the Y-SNP single base extensions amplicons were detected by ABI PRISM® 3100 Genetic Analyzer (Applied Biosystems) and analysed using GeneScan® Analysis Software Version 3.7 (Applied Biosystems).

We investigated locus DYS 393 and DYS456 by sequencing the region containing tetranucleotidic microsatellite (both AGAT), in order to confirm Y filer ambiguous results. We used a standard protocol of amplification, followed by sequencing with BigDye® Terminator v3.1 Cycle Sequencing Kit (Applied Biosystems) according to supplier's recommendations.
