# Supplementary material for: Analysis of Population Substructure in Two Sympatric Populations of Gran Chaco, Argentina
Source: PLoS One. 2013 May 22;8(5):e64054. doi: 10.1371/journal.pone.0064054 (PMC3661677; doi:10.1371/journal.pone.0064054)
Supplement: Text S2 — Methods for mtDNA Haplogroup assignation. (DOC) [file pone.0064054.s013.doc]

**Text S2 Methods for mtDNA Haplogroup assignation**

Mitochondrial DNA haplogroups were assigned by sequencing of about 1500 nt of the mtDNA control region (Behar et al., 2007), beginning at nucleotide position (np) 16000, followed by RFLP analysis as previously described (Torroni et al., 1995).

The sequence reaction was carried out with three different primers (Torroni et al., 1995) with the ABI Prism Big Dye Terminator Kit Sequencing (Applied Biosystems), then products were purified by ethanol precipitation and the DNA sequencing was performed on ABI PRISM® 3730 Genetic Analyzer (Applied Biosystems); ABI profiles were then analysed by SeqScape® Software v2.5 (Applied Biosystems) and compared to the revised Cambridge Reference Sequence (rCRS, NCBI: NC_012920.1 gi:251831 106) (Andrews et al, 1999).
