# Supplementary material for: Analysis of Population Substructure in Two Sympatric Populations of Gran Chaco, Argentina
Source: PLoS One. 2013 May 22;8(5):e64054. doi: 10.1371/journal.pone.0064054 (PMC3661677; doi:10.1371/journal.pone.0064054)
Supplement: Text S3 — Sororate definition. (DOC) [file pone.0064054.s014.doc]

**Text S3**. **Sororate definition.**

Sororate marriage is a social practice complementary to the levirate, that is usually associated with polygyny and patrilineal descent and patrilocal residence, in which a man marries the sister of his deceased wife. This kind of mating is the expression of the desire to keep the covenant established with the former marriage and to retain the related rights and obligations.
