# Supplementary figures and images for: Correction: Analysis of Population Substructure in Two Sympatric Populations of Gran Chaco, Argentina
Source: PLoS One. 2014 Jan 3;9(1):10.1371/annotation/623e9573-9757-42ba-96c3-870881faf06a. doi: 10.1371/annotation/623e9573-9757-42ba-96c3-870881faf06a (PMC3880417; doi:10.1371/annotation/623e9573-9757-42ba-96c3-870881faf06a)

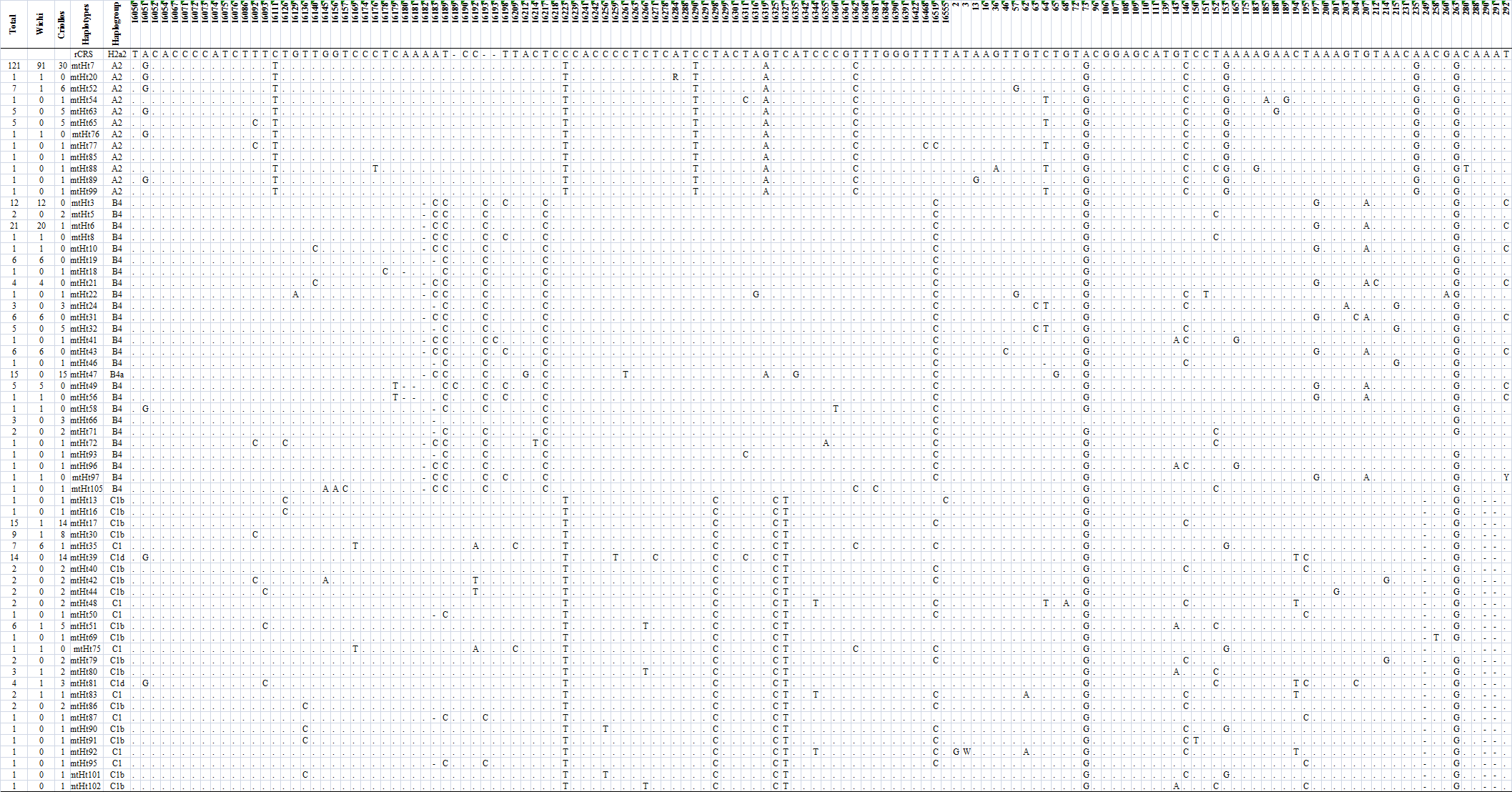


Table S6. Haplotypes observed in the two populations belonging to mtDNA haplogroups A2, B4 and C1.

Supplement: Supplementary file 1 [file pone.623e9573-9757-42ba-96c3-870881faf06a.s001.doc]

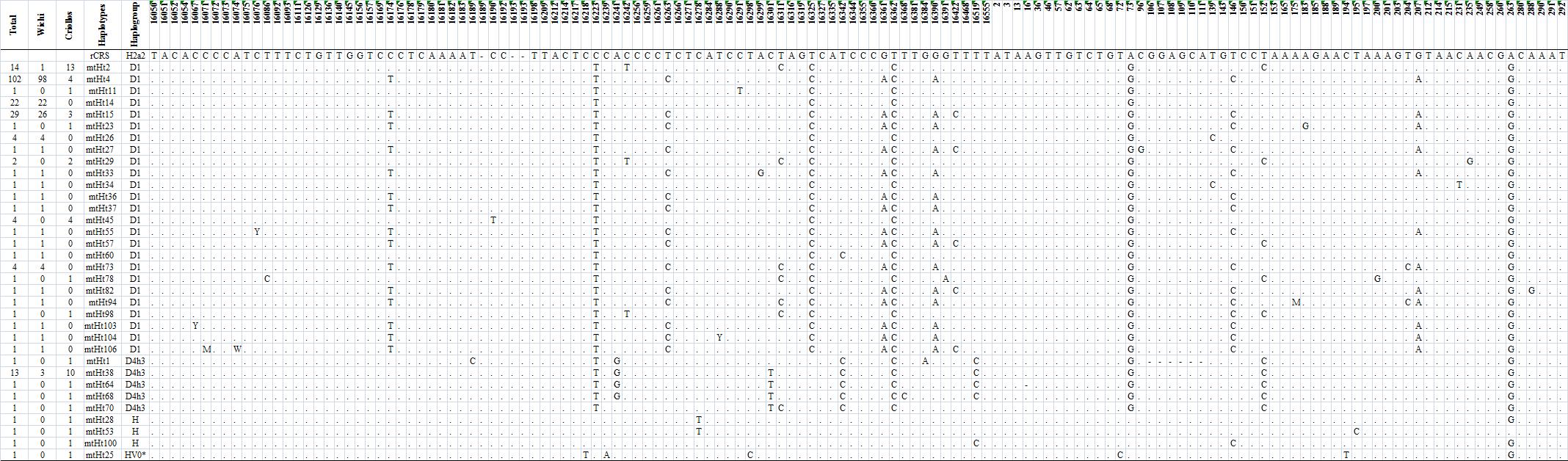


Table S7. Haplotypes observed in the two populations belonging to mtDNA haplogroups D1, H and HV0.

Supplement: Supplementary file 2 [file pone.623e9573-9757-42ba-96c3-870881faf06a.s002.doc]
